# The high-volume haemodiafiltration vs high-flux haemodialysis registry trial (H4RT): a multi-centre, unblinded, randomised, parallel-group, superiority study to compare the effectiveness and cost-effectiveness of high-volume haemodiafiltration and high-flux haemodialysis in people with kidney failure on maintenance dialysis using linkage to routine healthcare databases for outcomes

**DOI:** 10.1186/s13063-022-06357-y

**Published:** 2022-06-27

**Authors:** Fergus J. Caskey, Sunita Procter, Stephanie J. MacNeill, Julia Wade, Jodi Taylor, Leila Rooshenas, Yumeng Liu, Ammar Annaw, Karen Alloway, Andrew Davenport, Albert Power, Ken Farrington, Sandip Mitra, David C. Wheeler, Kristian Law, Helen Lewis-White, Yoav Ben-Shlomo, Will Hollingworth, Jenny Donovan, J. Athene Lane

**Affiliations:** 1 Population Health Sciences, Bristol Medical School, University of Bristol, Canynge Hall, 39 Whatley Road, Bristol, BS8 2PS UK; 2 Renal unit, Southmead Hospital, North Bristol NHS Trust, Bristol, BS10 5NB UK; 3 Bristol Trials Centre, 1-5 Whiteladies Road, Bristol Medical School, University of Bristol, Bristol, BS8 1NU UK; 4 Research and Innovation, Southmead Hospital, Bristol, BS10 5NB UK; 5 UCL Department of Renal Medicine, Royal Free Hospital, University College London, London, England; 6 Renal Unit, Lister Hospital, East and North Hertfordshire NHS Trust, Coreys Mill Lane, Coreys Mill Ln, Stevenage, SG1 4AB UK; 7 Renal Unit, Manchester University Hospitals NHS Trust, Manchester, UK; 8 George Institute for Global Health, Sydney, Australia; 9 Public and patient involvement representative, Bristol, UK

**Keywords:** H4RT, Kidney failure, Randomised controlled trial, Haemodialysis, Haemodiafiltration, Integrated qualitative research

## Abstract

**Background:**

More than a third of the 65,000 people living with kidney failure in the UK attend a dialysis unit 2–5 times a week to have their blood cleaned for 3–5 h. In haemodialysis (HD), toxins are removed by diffusion, which can be enhanced using a high-flux dialyser. This can be augmented with convection, as occurs in haemodiafiltration (HDF), and improved outcomes have been reported in people who are able to achieve high volumes of convection. This study compares the clinical- and cost-effectiveness of high-volume HDF compared with high-flux HD in the treatment of kidney failure.

**Methods:**

This is a UK-based, multi-centre, non-blinded randomised controlled trial. Adult patients already receiving HD or HDF will be randomised 1:1 to high-volume HDF (aiming for 21+ L of substitution fluid adjusted for body surface area) or high-flux HD. Exclusion criteria include lack of capacity to consent, life expectancy less than 3 months, on HD/HDF for less than 4 weeks, planned living kidney donor transplant or home dialysis scheduled within 3 months, prior intolerance of HDF and not suitable for high-volume HDF for other clinical reasons. The primary outcome is a composite of non-cancer mortality or hospital admission with a cardiovascular event or infection during follow-up (minimum 32 months, maximum 91 months) determined from routine data. Secondary outcomes include all-cause mortality, cardiovascular- and infection-related morbidity and mortality, health-related quality of life, cost-effectiveness and environmental impact. Baseline data will be collected by research personnel on-site. Follow-up data will be collected by linkage to routine healthcare databases — Hospital Episode Statistics, Civil Registration, Public Health England and the UK Renal Registry (UKRR) in England, and equivalent databases in Scotland and Wales, as necessary — and centrally administered patient-completed questionnaires. In addition, research personnel on-site will monitor for adverse events and collect data on adherence to the protocol (monthly during recruitment and quarterly during follow-up).

**Discussion:**

This study will provide evidence of the effectiveness and cost-effectiveness of HD as compared to HDF for adults with kidney failure in-centre HD or HDF. It will inform management for this patient group in the UK and internationally.

**Trial registration:**

ISRCTN10997319. Registered on 10 October 2017

**Supplementary Information:**

The online version contains supplementary material available at 10.1186/s13063-022-06357-y.

## Administrative information

Note: the numbers in curly brackets in this protocol refer to SPIRIT checklist item numbers. The order of the items has been modified to group similar items (see http://www.equator-network.org/reporting-guidelines/spirit-2013-statement-defining-standard-protocol-items-for-clinical-trials/).

**Table:** 

Title {1}	The High-volume Haemodiafiltration vs High-flux Haemodialysis Registry Trial (H4RT): a multi-centre, unblinded, randomised, parallel-group, superiority study to compare the effectiveness and cost-effectiveness of high-volume haemodiafiltration and high-flux haemodialysis in people with kidney failure on maintenance dialysis using linkage to routine healthcare databases for outcomes.
Trial registration {2a and 2b}.	ISRCTN10997319. Registered on 10^th^ October 2017. Prospectively registered.
Protocol version {3}	Version 8, 3 May 2022
Funding{4}	National Institute for Health Research (NIHR) Health Technology Assessment (HTA) Programme, project number 15/80/52
Author details {5a}	1 Population Health Sciences, Bristol Medical School, University of Bristol, Canynge Hall, 39 Whatley Road, Bristol, BS8 2PS, UK 2 Renal unit, Southmead Hospital, North Bristol NHS Trust, Bristol, BS10 5NB, UK 3 Bristol Trials Centre, 1-5 Whiteladies Road, Bristol Medical School, University of Bristol, Bristol, BS8 1NU, UK 4 Research and Innovation, Southmead Hospital, Bristol, BS10 5NB, UK 5 UCL Department of Renal Medicine, Royal Free Hospital, University College London, London, England 6 Renal Unit, Lister Hospital, East and North Hertfordshire NHS Trust, Coreys Mill Lane, Coreys Mill Ln, Stevenage, SG1 4AB, UK 7 Renal Unit, Manchester University Hospitals NHS Trust, Manchester, UK 8 George Institute for Global Health, Sydney, Australia 9 Public and patient involvement representative, Bristol, UK
Name and contact information for the trial sponsor {5b}	North Bristol NHS Trust, Southmead Hospital, Bristol, UK. BS10 5NB researchsponsor@nbt.nhs.uk
Role of sponsor {5c}	The sponsor played no part in study design and will play no part in the collection, management, analysis, and interpretation of data. The sponsor is however responsible for overall oversight of the trial. Drafts of all reports will be shared with the Sponsor for approval prior to submission for publication.

## Introduction

### Background and rationale {6a}

Kidney failure affects around 65,000 people in the UK, with more than 7000 newly affected people starting kidney replacement therapy (dialysis or kidney transplantation) each year [1]. It ranks among the most severe of the chronic non-communicable diseases — the survival probability at 1, 3 and 5 years is around 90, 70 and 50%, respectively [2] — and people on dialysis in the UK are admitted to hospital on average around 1.5–2.0 times per year [3]. Quality of life (QoL) is also well below that of the general population [4].

Currently, around 90% of existing dialysis patients are on some form of haemodialysis (HD) or haemodiafiltration (HDF) [1]. Although HD and HDF can be set-up at home, the majority is performed in-centre. HD and HDF cost approximately £25k per patient per year [5]. Treating the 25,000 people on HD/HDF costs around £500million of NHS spending each year [5], with a further £75million spent on hospital admissions and £50million on transport to and from dialysis [5].

HD relies on ‘diffusion’, a process by which molecules at high concentrations in the blood pass across a membrane in an artificial kidney (dialyser) to reach low concentrations in the dialysate fluid. Initially, membranes were designed with small pores to avoid the loss of essential proteins, meaning that only small-sized toxic molecules could leave the blood. As technology advanced, these pores became larger and asymmetrical, allowing larger toxic molecules to leave the blood whilst essential proteins were retained. These “high-flux” membranes are now recommended as standard practice in the UK [6]. HDF is similar to HD in that it uses diffusion to clean the blood, but at the same time, it uses ‘convection’ — a process that pulls fluid across the membrane, taking any dissolved solutes with it. Pulling large volumes of fluids across the membrane is considered ‘high-volume’ HDF. Adding convection achieves more efficient removal of middle-sized water-soluble molecules, with similar convective clearance of urea and β2 microglobulin. These larger uraemic molecules are thought to be responsible for some of the cardiovascular damage, impaired immunity and other organ damage associated with kidney failure [7]. This could explain why meta-analyses of existing randomised controlled trials indicate improved morbidity and mortality across a range of cardiovascular- and infection-related outcomes in patients achieving high-volume HDF [8–11].

The current standard of care, high-flux HD, is water intensive: each treatment requires approximately 500L of mains water to generate around 120L of dialysate water [12]. Given the exposure of the blood to such large quantities of water, it is important that this water contains safe levels of bacteria and endotoxins. As high-volume HDF involves infusing an additional 20–25L of water back into the patient 3 times per week, 52 weeks per year, the quality of water becomes even more crucial. Technological developments over the past decade now make it possible to produce such water ‘on-line’, i.e. continuously in the kidney unit, with filters built into dialysis machines to ensure it is sterile [13]. This shifts responsibility for water quality to individual kidney units and raises the importance of monitoring water quality if units are to provide a safe HDF service.

The other concern about high-volume HDF is that the removed fluid may contain important solutes and proteins (such as albumin) that are not replaced in the ultrapure substitution fluid. This could have an adverse impact on a patient’s nutritional status [14].

Three meta-analyses have compared different forms of HDF with different forms of HD and have drawn differing conclusions [9, 11, 15]. One found no beneficial effect of HDF on all-cause mortality but included very old studies of HDF regimens very different from current practice [11]; after removing these studies, the relative risk of mortality became 0.82 (95% CI 0.72–0.93) [11]. The other two found no evidence of the effect on all-cause mortality overall [9, 15]. However, the importance of HDF volume had not been appreciated when these trials were conceived and so trial practice varied widely between kidney units from low-volume to high-volume convection. A post hoc analysis of all the three recent major RCTs [16–18] found that patients achieving high-volume HDF (defined in the three trials as >25.4L, around 20.3L and >21.9L of convection per treatment) experienced a 39–46% lower risk of all-cause mortality [9]. This pooled data analysis also looked at specific causes of mortality and found similar effect sizes in cardiovascular- and infection-related deaths [9]. These findings informed the trial design in two ways:

- The intervention was defined as aiming for a substitution volume of greater than or equal to 21L adjusted for body surface area, equivalent to greater than or equal to 23L convection volume in a typical patient needing 2L of ultrafiltration to reach their target weight.
- The events for inclusion in the composite primary endpoint were chosen on the following bases:
  - ○ Non-cancer mortality — cancer would not be biologically expected to be affected by convection and cancer is more reliably captured as a cause of death in UK mortality data, compared with cardiovascular and respiratory infection [19].
  - ○ Hospital admission due to a cardiovascular event or infection — the pooled analyses of previous RCTs had shown lower deaths due to cardiovascular events and infection in people achieving high-volume HDF [9], and hospital episode data reliably captures events at this high level [20]

Despite the lack of evidence of clinical effectiveness, around 15% of patients in the UK were receiving HDF when this trial was being planned, with wide variation in HDF adoption and plans for further adoption between centres (unpublished UKRR data, Oct 2015).

The average phase III efficacy trial in the USA is estimated to cost $19 million [21], which risks restricting this level of evidence generation to treatments with the potential to re-coup such costs. There is therefore a need to find novel ways to generate such evidence more efficiently [22], and in 2015, the National Institute for Health (NIHR) Research Health Technology Assessment in the UK put out a first call for efficient trials. This study, funded through this scheme and using an efficient trial design with linkage for outcomes data, aims to establish the effectiveness and cost-effectiveness of high-volume HDF compared with high-flux HD in adult patients with kidney failure on maintenance, in-centre HD or HDF at least three times a week.

### Objectives {7}

The primary objective is to determine the relative effectiveness of high-volume HDF compared with high-flux HD on non-cancer mortality and hospital admission due to a cardiovascular event or infection.

The secondary objectives are to determine the relative effectiveness of high-volume HDF compared with high-flux HD on:

- All-cause mortality
- Cardiovascular events associated with death
- Cardiovascular events associated with hospital admission
- Infection events associated with death
- Infection events associated with hospital admission
- Health-related quality of life
- Cost-effectiveness
- Environmental impact
- Water quality testing and breaches

### Trial design {8}

**Fig. 1:**
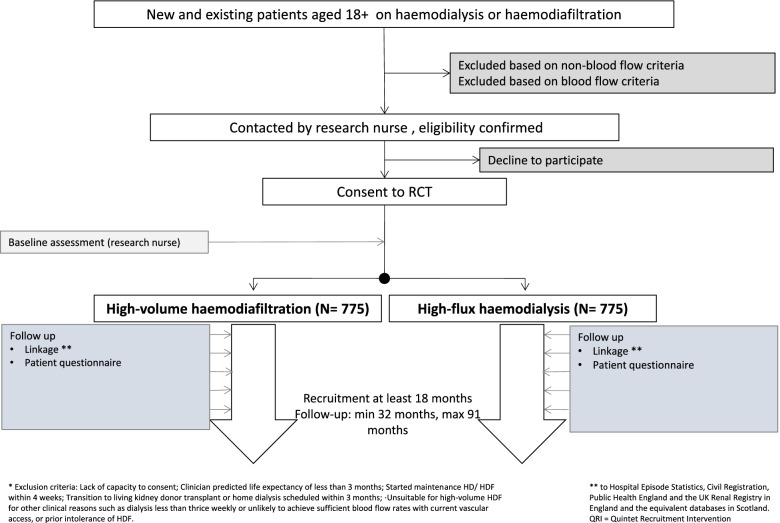
The High-volume Haemodiafiltration vs High-flux Haemodialysis Registry Trial

This is a non-blinded, randomised, parallel-group, controlled trial comparing high-volume HDF (aiming for 21+L of substitution fluid adjusted for body surface area) against high-flux HD, randomised 1:1 and stratified by site, age group (18–64 and 65+) and residual kidney function (urine volume < 100mL/day and 100+ mL/day [23–25], using linkage to routine healthcare databases for outcomes (Fig. 1). A QuinteT Recruitment Intervention (QRI) is integrated into the initial 18 months of trial recruitment to optimise recruitment and informed consent to the trial [26]. A health economic evaluation is exploring the cost-effectiveness of high-volume HDF during the follow-up period from an NHS perspective. Additional analyses will explore any impact of treatment on residential care. Should differences in any component of the primary outcome between the study groups be evident at the end of trial, resulting in uncertainty over the longer-term cost-effectiveness of high-volume HDF, we will develop a decision analysis model to extrapolate cost-effectiveness estimates over patient lifetimes.

## Methods: participants, interventions and outcomes

### Study setting {9}

All 65 adult kidney units providing dialysis services in England, Scotland and Wales are being invited to participate. At least 33 are expected to participate. Information governance arrangements in Northern Ireland at the time of trial set-up will mean that kidney units there cannot participate, given the need for linkage to routine data for trial outcomes. A full list of study sites is available on the trial website [27].

### Eligibility criteria {10}

The inclusion criteria for participants:

- Being aged 18 years or over and in receipt of in-centre, maintenance HD or HDF for kidney failure
- Dialysing at least three times a week in a main dialysis or satellite unit
- Having the potential to achieve high-volume HDF

The exclusion criteria for participants:

- Lacking capacity to consent
- Having a prognosis of less than 3 months
- Having started maintenance HD or HDF within the preceding 4 weeks
- Having a transition to living kidney donor transplant or home dialysis scheduled within the next 3 months
- Being unsuitable for high-volume HDF for other clinical reasons such as dialysis less than thrice weekly or unlikely to achieve sufficient blood flow rates with current vascular access, or prior intolerance of HDF

### Who will take informed consent? {26a}

Consent is being received by researchers trained in Good Clinical Practice (GCP). Consent to participate in the qualitative interviews is being received by qualitative researchers with GCP training.

### Additional consent provisions for collection and use of participant data and biological specimens {26b}

Consent includes permission to process data through linkage. The full lawful bases for processing all personal and sensitive data for the study are set out in the *Trial*’s privacy notice [28].

## Interventions

### Explanation for the choice of comparators {6b}

The comparator is in-centre, high-flux HD, which is usually delivered for around 4 h three times a week. It requires ultrapure water (bacterial limit < 0.1 colony-forming units (CFU) per mL; endotoxin limit < 0.03 endotoxin units (EU) per mL). The small solute-based dialysis sessional adequacy target is the same as for the HDF arm (a single pool Kt/V ≥ 1.4, as recommended in national guidelines) [6].

### Intervention description {11a}

The intervention is in-centre, high-volume HDF which is usually delivered for around 4 h three times a week aiming for a sessional 21+ L of substitution fluid per 1.73m^2^ body surface area [29, 30], adjusted pro rata for patients dialysing more than three times a week. This requires sterile water (bacterial limit < 10^−6^ CFU per mL; endotoxin limit < 0.03 EU per mL). As for the high-flux HD arm, a small solute-based dialysis sessional adequacy of single pool Kt/V ≥ 1.4 is being targeted.

### Criteria for discontinuing or modifying allocated interventions {11b}

Participants can discontinue their allocated treatment if they or their clinical team decide that the randomised treatment is no longer clinically appropriate, based on symptoms or blood results. A participant will be considered to have deviated from their allocated treatment if:

1. They are not on that allocated treatment for the last dialysis session of two consecutive months (i.e. allocated to HDF but receiving HD, or vice versa)
2. They and their clinical team are not aiming for high-volume HDF for two consecutive months. (this applies to the HDF-only arm)

### Strategies to improve adherence to interventions {11c}

A standard operating procedure providing instructions on improving adherence through appropriate choice of dialysis needle gauge and blood pump speed (and therefore attaining the target substitution volume) has been developed by the investigators. Sites will provide the trial unit with reports on compliance monthly during recruitment and three-monthly during follow-up; the trial unit will analyse these data and feedback to the research nurses and PI in each site monthly during recruitment and three-monthly during follow-up. Training in achieving high-volume HDF will be on-going.

Adherence to the protocol will be logged monthly by the clinical team throughout recruitment and 3-monthly throughout the follow-up and submitted to the trial unit. For the purposes of reporting, only the last recorded dialysis session of each month will be examined. The log will include receipt of the allocated modality, attainment of high-volume HDF (if applicable) and water quality testing. Deviation from the protocol has been defined as (a) being off their allocated treatment on the last dialysis session of the month for two consecutive months and (for those allocated to high-volume HDF) (b) no longer aiming for high-volume HDF. This definition recognises that some patients need time to adjust their dialysis prescription and achieve their target substitution volume; indeed, some, despite all best efforts, may never achieve it. It also recognises that patients may have short periods off their allocated treatment due to admission to a hospital or travel with dialysis in another centre.

At the end of the trial, a data extract will also be requested from sites and the UKRR to explore adherence at the dialysis session level.

### Relevant concomitant care permitted or prohibited during the trial {11d}

Industry partners continue to work on dialyser technology, and whether these innovations can be permitted as concomitant care will need to be considered on a case-by-case basis. As a rule, however, middle and high cut-off dialysers cannot be used by participants in either arm of the trial. First, they are not compatible with delivering HDF. Second, they may attenuate any treatment effect of high-volume HDF if they increase the removal of middle molecules when used to deliver HD, as they claim.

Although patients should not be included in the trial if transition to a living kidney donor transplant or home dialysis is scheduled within the next 3 months, patients may receive a kidney transplant or choose to transition to home therapy at any time during follow-up, if this is considered the most appropriate treatment for them in discussion with their clinical team.

### Provisions for post-trial care {30}

If there is negligent harm during the trial when the NHS body owes a duty of care to the person harmed, NHS indemnity covers NHS staff, medical academic staff with honorary contracts and those conducting the study. NHS indemnity does not cover no-fault compensation and is unable to agree in advance to pay compensation for non-negligent harm.

Following completion of the trial, the choice of on-going kidney replacement therapy modality will be a shared informed decision between the patient and the kidney unit.

### Outcomes {12}

#### Primary outcome measure

The primary outcome is a composite of first of non-cancer mortality or admission to hospital related to a cardiovascular event or infection. The outcome will be identified using routine Hospital Episode Statistics and Civil Registration data, and their equivalents in Scotland and Wales, as necessary.

Secondary outcome measures are:

- All-cause mortality (source: trial data, civil registration and UKRR)
- Non-cancer mortality (source: civil registration)
- Cardiovascular — cause-specific hospitalisation and mortality (source: hospital statistics and civil registration)
- Infection — cause-specific hospitalisation and mortality (source: hospital statistics and civil registration) and reportable infections (MRSA and MSSA) (source: Public Health England to September 2021, UK Health Security Agency from October 2021)
- Health-related quality of life — preference-based quality of life derived from EQ-5D-5L, disease-specific quality of life (Dialysis Symptom Index) and time to recover after each dialysis [4] (source: patient questionnaires)
- Indirect effects: routinely measured/prescribed and recorded anaemia disorder management (haemoglobin levels and erythropoiesis-stimulating agent dose), mineral bone disorder management (calcium, phosphate and PTH levels and phosphate binder dose) and nutritional status (albumin level) (source: UKRR and data extracts from electronic health records)
- Cost-effectiveness from an NHS perspective (source: UKRR, hospital statistics for in-patient and out-patient activity and patient questionnaires for primary care, community and residential care)
- Impact on the environment, including locally purified water, manufactured saline and plastic consumables

Assessments and follow-up: The components and timings of follow-up measures are shown in Table 1.

**Table 1:** Assessments and follow-up

Procedures	Data	Screening	Baseline	Treatment phase		Event based
			Face-to-face visit 1	Follow-up (min = 32 month, max = 91 months) No visits		
				Linkage	Patient questionnaire (6 monthly)	
Eligibility assessment		√				
Informed consent			√			
Randomisation			√			
Demographics	Age, sex, ethnicity, marital status, education level, smoking history		√			
Clinical (1)	Primary renal disease, date first seen by a nephrologist, co-morbidities, dietary restrictions, 24-h urine volume		√			
Clinical (2)	RRT treatment history, prescribed medication (including erythropoiesis-stimulating agents and phosphate binders)		√	√		
Physical assessment (1)	Height, heart rate		√			
Physical assessment (2)	Weight, systolic and diastolic blood pressure		√	√		
Resource use (1)	Day case and inpatient hospital admissions (including surgical procedures performed)		√	√		
Resource use (2)	Nursing home/residential home days/hospice days, other hospital out-patient services and primary care and community services in the last 6 months		√		√	
Laboratory tests	Creatinine, urea, Kt/V, urea reduction ratio, albumin, haemoglobin, haematocrit, mean corpuscular volume, sodium, potassium, bicarbonate, corrected calcium, phosphate, C-reactive protein, intact parathyroid hormone, total cholesterol. (From the date of the study visit or the closest date prior to the study visit)		√	√		
Patient reported	EQ-5D-5L and DSI and time to recovery [31]		√		√	
SAE reporting						√

### Participant timeline {13}

See Fig. 1 for the participant’s timeline through the trial.

### Sample size {14}

Based on data from the UKRR [2] and prior linkage of the UKRR to Hospital Episode Statistics [3], we anticipate that at 3 years of follow-up 65% of patients on HD will have experienced our composite endpoint and we plan to detect a hazard ratio (HR) of 0.75. This effect size was agreed to be clinically significant at an investigator meeting involving patients and healthcare professionals. We assume, however, that any effect will be attenuated by (i) cross-over between arms (15% HD to HDF and 5% HDF to HD) and (ii) participants being allowed to take part in other trials simultaneously. To optimise recruitment and avoid excluding eligible patients because they are already participating/want to participate in other trials, an additional adjustment has been made. The latter assumes that up to half of patients in both groups will take part in another trial that assigns half of these to an intervention that reduces our composite endpoint (HR = 0.9). We therefore anticipate the proportion experiencing an event on high-flux HD will be 62.5% (37.5% surviving event-free) and on HDF it will be 54.1% (45.9% surviving event-free) giving a revised HR for the H4RT study of 0.79. The number of events required to detect this difference with 90% power and a 5% significance level is 801, which requires 1348 participants in total. The primary analysis will be intention to treat (ITT), and to avoid informative censoring, participants will not be censored at the time of kidney transplantation or transition to home dialysis. Allowing for 10% loss to follow-up for other reasons, we require 1527 participants and will recruit 1550.

### Recruitment {15}

Identification, screening and consent procedures will be undertaken by research staff and treating clinicians who will be trained and competent to participate according to the ethically approved protocol, principles of GCP and the Declaration of Helsinki. It will take place in several steps:

1. Nurses in dialysis units will provide a list of potentially eligible patients in their units
2. Eligibility will be confirmed by the patient’s treating clinician or the local principal investigator
3. Standard letters will be sent out/handed out to potentially eligible patients introducing the study and including a patient information sheet
4. Letters will be followed up with a telephone call/face-to-face visit from the research staff to offer further discussion about the study/a baseline visit at a scheduled dialysis attendance
5. Potentially eligible patients will be approached according to their regular dialysis shift
6. Potential participants will collect their urine for 24 h (or inter-dialytic) prior to the recruitment visit, as a 24-h urine volume is required to conduct randomisation as one of the stratification variables. (If a 24-h urine volume is available from the 6 weeks prior to randomisation, this can be used/does not need repeating.) Randomisation will take place once this information is available and the participant and their dialysis nurses informed
7. Each potential participant will be asked to provide written informed consent to be randomised to high-volume HDF or high-flux HD, and followed up through their routine health records and patient questionnaires. The patient information sheet and the consent form will explain the need for long-term follow-up and linkage to other routine health databases
8. Patients who are not willing to be randomised, but who would otherwise be eligible, will be asked to consent to other research (e.g. interviews to explore their views on the quality of information provided about the trial, and how they reached their decision about participation)

Anticipating the challenges to recruitment to this trial, funding was sought and approved to integrate the QRI into the trial from the set-up stage. This intervention has been designed to support recruitment and informed consent to trials, particularly those anticipated to be challenging for recruitment [26].

The QRI will proceed in two iterative phases: sources of recruitment difficulties are rapidly investigated in phase I, informing a mix of standardised and tailored interventions to improve recruitment in phase II. During phase 1, specific or wider recruitment obstacles will be identified using a combination of data sources including trial documentation, screening log data, interviews with professionals involved in delivering the trial and patients invited to participate, audio-recordings of recruitment consultations and observations of trial management group (TMG) meetings. If recruitment difficulties are evident across the study or in particular centres, the QRI team will work closely with the TMG/CI to formulate a ‘plan of action’ that intends to improve recruitment and information provision. The components of this plan will be grounded in the findings from phase 1 and may include study-wide, centre-specific or individual recruiter-level interventions to optimise recruitment.

## Assignment of interventions: allocation

### Sequence generation {16a}

Participants will be randomly assigned to the high-volume HDF or high-flux HD with a 1:1 allocation as per a computer-generated randomisation schedule stratified by site, age (18–64 years and ≥ 65 years) and residual kidney function (urine volume < 100 mL/day and ≥ 100 mL/day) using permuted blocks of random sizes. The block sizes will not be disclosed, to ensure concealment. Participants will primarily be randomised using an online, central randomisation service. This service can also be accessed by telephone if Internet access is limited, for example recruiting off-site.

### Concealment mechanism {16b}

The web-based and telephone randomisation system ensures allocation concealment. The service will not release the randomisation code until the patient has been recruited into the trial, which takes place after all baseline measurements have been completed.

### Implementation {16c}

The allocation sequence generation is embedded in the web-based and telephone system. Research nurses based at sites enrol participants and randomise them using the web-based or telephone randomisation system.

## Assignment of interventions: blinding

### Who will be blinded {17a}

Due to the nature of the intervention, participants and those administering the intervention will not be blinded to group allocation. Two statisticians are supporting this trial. The senior statistician co-applicant will remain blinded throughout the trial. The trial statistician will perform all disaggregated analyses according to a pre-specified statistical analysis plan and attend closed Data Monitoring Committee (DMC) meetings, as required.

### Procedure for unblinding if needed {17b}

There is no requirement for unblinding.

## Data collection and management

### Plans for assessment and collection of outcomes {18a}

Baseline clinical and patient-reported data will be collected by research nurses (following consent and prior to randomisation) (Table 2). Validated questionnaires will be used for patient-reported outcomes.

**Table 2:** Summary of baseline data collection for the randomised controlled trial

Demographics/social	Age, sex, ethnicity, marital status, education level, smoking history
Clinical	Primary renal disease, date first seen by a nephrologist, renal replacement therapy history, co-morbidities, prescribed medication (including erythropoiesis-stimulating agents and phosphate binders), 24-h urine volume (within the 6 weeks preceding randomisation)
Resource use	Day case and inpatient hospital admissions (including surgical procedures), nursing home/residential home days/hospice days, other hospital out-patient services, and primary care and community services in the last 6 months
Laboratory	Creatinine, urea, Kt/V, urea reduction ratio, albumin, haemoglobin, haematocrit, mean corpuscular volume, sodium, potassium, bicarbonate, corrected calcium, phosphate, C-reactive protein, intact parathyroid hormone, total cholesterol (from the date of the study visit or the closest date prior to the study visit)
Physical assessment	Height, weight, blood pressure, heart rate
Patient reported	EQ-5D-5L, Dialysis Symptom Index and time to recovery [31]

Follow-up will continue for a minimum of 32 months and a maximum of 91 months. It will be undertaken through a combination of 6-monthly patient questionnaires and by linkage to routine healthcare databases — Hospital Episode Statistics, Civil Registration, Public Health England (PHE) to September 2021 and UK Health Security Agency (UKHSA) from October 2021, and the UKRR in England, and the equivalent databases in Scotland and Wales, as necessary (Table 3). Only data that are collected as part of routine care will be collected. Paper and electronic (web portal) options will be offered to patients for patient questionnaire completion.

**Table 3:** Summary of follow-up data collection

	Data items	Source
Routine laboratory data	Creatinine, urea, Kt/V, urea reduction ratio, albumin, haemoglobin, haematocrit, mean corpuscular volume, sodium, potassium, bicarbonate, corrected calcium, phosphate, C-reactive protein, intact parathyroid hormone, total cholesterol	UKRR
Cardiovascular and infection hospital admission data	Cardiovascular: nonspecific chest pain (102); congestive heart failure, non-hypertensive (108); coronary atherosclerosis (101); other circulatory diseases (117); acute myocardial infarction (100); peripheral and visceral atherosclerosis (114); chronic ulcer of skin (199); Gangrene (248); aortic, peripheral and visceral arterial disease (115); transient cerebral ischemia (112); cardiac arrest and ventricular fibrillation (107); pulmonary heart disease (103); other and ill-defined cerebrovascular disease (111); acute cerebrovascular disease (109) Infection: pneumonia (122); septicemia (except in labour) (2); pleurisy, pneumothorax, pulmonary collapse (130); aortic and peripheral arterial emboli (116); tuberculosis (1); mycoses (4); HIV infection (5); encephalitis (77); meningitis (76); shock (249); skin and subcutaneous tissue infection (197); fever of unknown origin (246); infective arthritis and osteomyelitis (201); bacterial infection, unspecified site (3); other inflammatory conditions of the skin (198); other infections, including parasitic (8); influenza (123); urinary tract infections (159); genitourinary symptoms and ill-defined conditions (163)	Hospital Statistics (HES, PEDW, ISD),
Mortality data	Non-cancer mortality (i.e. all causes of death excluding chapter II causes in ICD-10)	NHS Spine tracing, UKRR, Hospital Statistics, Civil Registration
Patient-reported outcomes	EQ-5D-5L, DSI and time to recovery (following dialysis) [31]	Patient questionnaire administered 6 monthly
RRT use	Frequency, machine, dialyser, dialysis times and consumables used	Annual census (extracted from the renal IT system)
Other hospital admissions	Day case and inpatient hospital admissions (including surgical procedures performed)	Hospital Statistics (HES, PEDW, ISD)
Patient-reported healthcare use	Nursing home/residential home days/hospice days, and primary care, community services and medication usage in the last 6 months	Patient questionnaire administered 6 monthly
Blood stream infections	Methicillin-resistant *Staphylococcus aureus*, methicillin-sensitive *Staphylococcus aureus*, *Clostridium difficile*, and *Escherichia coli*, as the report by mandate to PHE/the UKHSA	Reported to PHE/UKHSA and shared with UKRR

Numbers in parentheses following diagnoses refer to the Healthcare Cost and Utilisation Project Clinical Classification System for mapping diagnoses onto ICD-10 www.hcup-us.ahrq.gov. *Abbreviations*: *HES* Hospital Episode Statistics, *ICD* International Classification of Diseases, *ISD* Information Services Division, *PEDW* Patient Episode Database for Wales, *PHE* Public Health England, *RRT *Renal Replacement Therapy, *UKHSA* UK Health Security Agency, *UKRR* UK Renal Registry

Adherence to the protocol will be monitored through monthly reports from sites during recruitment, reducing to three-monthly during follow-up. As the UKRR follows all patients on kidney replacement therapy in the UK, patients should not be lost to follow-up unless they move to another country or opt out of data linkage after being randomised.

### Plans to promote participant retention and complete follow-up {18b}

As the primary outcome relies on data linkage with national routine healthcare databases, participants will only be lost to follow-up if they opt out of data linkage after being randomised. Patients who discontinue allocated treatment can choose to continue to receive questionnaires and allow data linkage. The minimum number of identifiers will be used to link with routine data sources, as agreed with those organisations and set out in the participant information sheet. For patient-reported outcomes, up to three reminders are sent.

### Data management {19}

Baseline data will be collected on paper by the local research nurses. Forms will be copied, with originals stored locally and copies transferred securely in tamper-proof envelopes to the trial unit for entry into the database. Staff at the trial unit will mail or email out the follow-up patient questionnaires, according to the participant’s stated preference, and enter the returned questionnaires into the database. Study data will be collected and managed using REDCap hosted at the University of Bristol. The database incorporates data entry and validation rules to reduce data entry errors and management functions to facilitate auditing and data quality assurance.

**Fig. 2:**
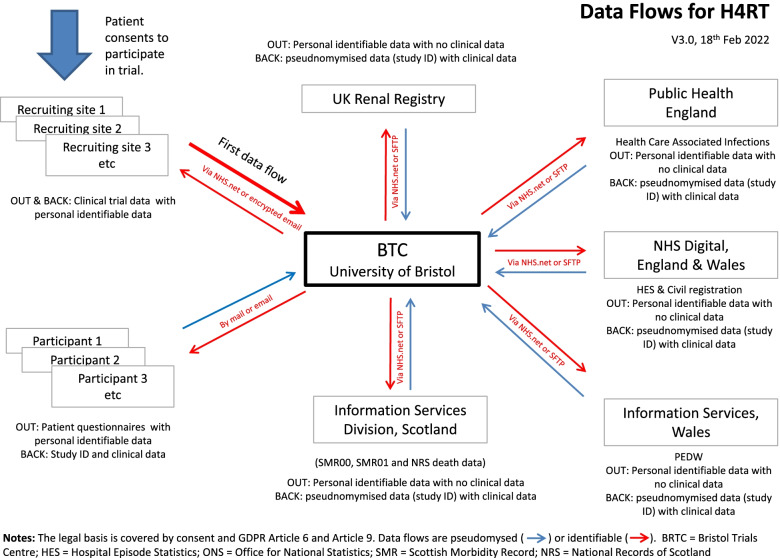
Data flows for H4RT

Identifiable information, as agreed with partner organisations and set out in the participant information sheet, will be used to link this primary dataset with existing routine healthcare databases for follow-up. The flow of data — identifiable and pseudonymised — is summarised in Fig. 2. The database system will protect patient information in line with the data protection legislation and any specific requirements of the partner organisations. Trial staff will ensure that participants’ anonymity is maintained through protective and secure handling and storage of patient information. The chief investigator (CI) will act as custodian of the full dataset.

### Confidentiality {27}

Personal identifiable and clinical data will be processed in compliance with the Common Law Duty of Confidentiality and Data Protection Act 2018, as set out in the privacy notice on the trial website [27].

### Plans for collection, laboratory evaluation and storage of biological specimens for genetic or molecular analysis in this trial/future use {33}

No biological specimens are being collected.

## Statistical methods

### Statistical methods for primary and secondary outcomes {20a}

All analyses and reporting will be in line with Consolidated Standards of Reporting Trials (CONSORT) guidelines. Primary analyses will be conducted on an ITT basis. A full statistical analysis plan will be developed and agreed by the trial steering committee (TSC) prior to undertaking the analyses of the main trial.

Descriptive statistics will be used to determine whether there are imbalances at baseline between treatment groups and will inform any later sensitivity analyses where appropriate additional adjustment will be performed. Patient-reported outcome scores based on standardised questionnaires will be calculated based on the developers’ scoring manuals and missing and erroneous items will be handled according to these manuals. Continuous measures will be presented as means and standard deviations or medians and ranges depending on their distribution. Categorical data will be presented as frequencies and proportions.

The primary outcome is a composite outcome of non-cancer death or hospital admission for infection or cardiovascular event by a minimum of 32 to a maximum of 91 months of follow-up. We will compare the distribution of time-to-events between the two groups using Kaplan-Meier curves and log-rank test. We will use Cox’s proportional hazards model — or an alternative flexible parametric model if the assumption of proportional hazards is not met — to compare the time-to-events between the two groups with adjustment for stratification variables.

The secondary outcomes of all-cause mortality, non-cancer mortality, cardiovascular mortality and infection mortality will be analysed and reported in a similar manner to the primary outcome. Cardiovascular- and infection-related hospitalisations and MRSA and MSSA infections are all recurrent events and analyses of such outcomes should account for informative censoring due to death. We will therefore use joint frailty models as these simultaneously analyse recurrent events (infections or hospitalisations) and time to death whilst estimating distinct hazard ratios. Appropriate repeated measures regression models for patient-reported outcomes will be chosen based on the distribution of the data and will adjust for stratification variables and values of the outcome at the time of randomisation. A similar repeated measures regression approach will be taken to the following other repeated measure outcomes: time to recover after each dialysis session, haemoglobin levels, erythropoiesis-stimulating agent dose, calcium levels, phosphate levels, PTH levels, albumin levels and phosphate binder dose.

All analyses will be adjusted for stratification variables and, in the case of patient-reported outcomes, we will also adjust for the value of the outcome pre-randomisation. For all statistical models used, the underlying assumptions will be checked using standard methods. If assumptions are not valid, then alternative methods of analysis will be sought.

### Interim analyses {21b}

A formal interim analysis of all-cause mortality will be conducted using available data and discussed with the DMC. A one-sided *p*-value < 0.025 for treatment differences (mortality (HD) < mortality (HDF)) would be considered a meaningful difference to be explored further. The analysis will take place half-way through the study although the exact timing of the analysis will be set by the DMC who will be guided by recruitment patterns and progress in obtaining permissions for relevant linkages.

### Methods for additional analyses (e.g. subgroup analyses) {20b}

We will conduct pre-planned subgroup analyses to investigate any differential treatment effects according to baseline urine volume (a surrogate for residual kidney function), diabetic status, line, weight at baseline, age and the experience of sites in achieving high-volume HDF. Subgroup analyses will be conducted by including an interaction term between allocated treatment and baseline characteristic in regression models then using the likelihood ratio test comparing this model with one excluding the interaction term. Urine volume and age will be included in the model as continuous variables.

A per-protocol analysis will be performed, with patients censored at the time of discontinuation of allocated treatment (including receipt of a kidney transplant, transition to home dialysis, receipt of the alternative form of HD/HDF on the last session of two sequential months and patient choice to come off allocated treatment), with adjustment made for baseline characteristics. Recognising biases inherent to per-protocol analyses when compliance is not random, we will also consider appropriate alternative causal inference approaches to estimate the treatment effect in compliers.

We will also test the sensitivity of the primary analysis to the definition of high-volume HDF (21+L of substitution volume, adjusted for body surface area) by testing two alternative definitions, (a) a lower threshold of 18.9+L of substitution volume, i.e. 10% below the original threshold, and (b) an absolute substitution volume of 21L, i.e. not adjusting for body surface area.

The primary economic analysis will take an NHS perspective in order to minimise the participant burden and increase the efficiency of the RCT. Additional analyses will explore any impact of treatment on residential care. The analysis will include a ‘within trial’ analysis estimating the cost-effectiveness of high-volume HDF during the trial follow-up period. The costs associated with high-flux HD and high-volume HDF will be determined by calculating the incremental cost of equipment, staff time and materials/consumables used in performing dialysis. Healthcare resource use, including hospital visits and admissions, GP and community care contacts and medication use will be obtained from linked hospital statistics in England, Scotland and Wales, as necessary, the UK Renal Registry, and patient self-completed questionnaires and evaluated using published unit cost sources [32–34].

QALYs will be estimated from EQ-5D-5L responses and mortality data during follow-up, adjusted for any baseline differences in EQ-5D-5L scores [35]. We will explore reasons for data missingness and, if necessary, data will be imputed using appropriate methods [36]. Cost and QALY data will be combined to calculate an incremental net monetary benefit (INMB) statistic [37]. The primary health economic analysis will estimate whether HDF is cost-effective at the established NICE threshold of £20,000 per QALY gained. Uncertainty in the point estimate of cost per QALY will be quantified to calculate confidence intervals around the INMB. The probability that HDF is cost-effective at various ‘willingness to pay’ thresholds and in the pre-specified subgroups described above (e.g. residual kidney function and age) will be assessed using a cost-effectiveness acceptability curve.

Should differences in any component of the primary outcome between the study groups be evident at the end of trial follow-up resulting in uncertainty over the longer-term cost-effectiveness of high-volume HDF, we will develop a probabilistic decision analysis model to extrapolate cost-effectiveness estimates over patient lifetimes. Further details of the economic analysis will be provided in a publicly available health economic analysis plan.

### Methods in analysis to handle protocol non-adherence and any statistical methods to handle missing data {20c}

Where missing data exist, the frequency of missing data will be indicated and if the amount of missing data differs substantially between treatment groups (>10%) potential reasons will be explored. Sensitivity analyses will be conducted (including the use of multiple imputation methods where assumptions are met) to examine the influence of missing data on the key trial findings.

### Plans to give access to the full protocol, participant-level data and statistical code {31c}

The full protocol is available as a supplement (Additional file 1). External groups will be able to apply to the trial management group to request access to anonymised patient-level data, as permitted by the data sharing agreements for data that has been provided through linkage.

## Oversight and monitoring

### Composition of the coordinating Centre and trial steering committee {5d}

The sponsor will be responsible for overall oversight of the trial. The study is supervised by a TMG consisting of grant holders and other relevant trial delivery staff. A TSC oversees the progress of the trial, comprising of an independent chair and five other independent members, including a public and patient involvement (PPI) representative, and the CI. A DMC monitors accumulating trial data for quality, completeness and patient safety and comprises of an independent chair and five other independent members and the CI.

### Composition of the data monitoring committee, its role and reporting structure {21a}

The DMC will monitor the trial every 6–12 months, depending on the phase of the trial, either in person or electronically. It includes an independent chair and independent members with clinical and methodological expertise. It reports to the chair of the TSC. The DMC charter is available from the corresponding author.

### Adverse event reporting and harms {22}

Given the intensive monitoring of dialysis patients in routine clinical care, the comprehensive data on clinical events recorded directly by the trial unit, and the routine use of both high-volume HDF and high-flux HD as part of routine NHS care, the study will utilise the following risk-adapted safety reporting approach:

1. Adverse events (AEs) will be regularly screened: (a) monthly during recruitment and quarterly during follow-up by the local research team and (b) as directed by the DMC using data collected specifically for the trial and data derived from linkage to routine healthcare databases.
2. Events not considered to be directly related to high-volume HDF, i.e. AEs and SAEs, do not require to be recorded and reported (using the standard reporting form) to the sponsor by the PI.
3. Events considered to be directly related to high-volume HDF but not serious, i.e. AEs, also do not require to be recorded and reported (using the standard reporting form) to the sponsor by the PI.
4. Events considered to be serious and directly related (or possibly/probably directly related) to high-volume HDF, i.e. serious adverse reactions (SARs) and suspected unexpected serious adverse reactions (SUSARs), do require reporting (using the standard reporting form) to the CI and the sponsor. The local research team should maintain a log of SAEs that they identify and agree are not SARs. This will not need to be routinely submitted to the CI, but will act as a local record of decision-making and may be asked for if a site is monitored.
5. Line listings of SAEs and reported SARs and SUSARs will be reviewed monthly by the CI and submitted annually to the DMC and REC.

### Frequency and plans for auditing trial conduct {23}

The study will be monitored in accordance with North Bristol NHS Trust’s Monitoring standard operating procedure. All trial-related documents will be made available on request for monitoring and audit by North Bristol NHS Trust, the Research Ethics Committee (REC), and available for inspection by other licensed bodies. Monitoring and audits undertaken by North Bristol NHS Trust, under their remit as sponsor, or individuals appointed with responsibility for monitoring on behalf of the Trust, will ensure adherence to GCP and the NHS Research Governance Framework for Health and Social Care (2nd edition). Remote monitoring will be conducted based on information submitted by sites and analysis of the trial database. Site visits will be initiated using a risk-based approach.

### Plans for communicating important protocol amendments to relevant parties (e.g. trial participants, ethical committees) {25}

All changes to the protocol will seek approval from the sponsor, REC, Health Research Authority and site research and development offices before local implementation. As judged necessary by the sponsor and REC, these changes will be communicated to the participants.

## Dissemination plans {31a}

The results of the study will be published in the academic press and presented at national and international conferences. The investigators will work with guideline writing organisations to ensure that they are aware of the new data and encouraged to incorporate these data into updates on their guidance. Working with the patient advisory group, the investigators will develop plain English summaries of the results of the trial for sharing with trial participants and disseminating to patients and the public more widely.

## Discussion

Outcomes on dialysis are as poor as many cancers [38] and there is a need to improve treatment options for people with kidney failure who are waiting for, or unsuitable for, kidney transplantation. A promising innovation has been the introduction of an element of convection in the form of HDF [39]. Meta-analyses have not, however, established the superiority of either treatment over the other [9, 11, 15]. One RCT did report a survival advantage of HDF over HD, but questions have been raised about the validity of this study as 10% of participants were removed from the HDF arm after randomisation due to low blood flows. Low blood flow is known to be associated with higher risk patients, as demonstrated by the baseline differences between the two arms in terms of diabetes and dialysis catheter use [17].

Post hoc analyses of recent trials have therefore looked at the dose of HDF to see if under-dosing might explain the previously null results. In patients who achieve high-volume HDF (varyingly defined, but generally requiring more than 20 L of convection per treatment), lower all-cause mortality was observed [8]. This appeared to be across cardiovascular and infection deaths. However, as patients had not been randomly allocated to receive high as opposed to low volumes of HDF, what they received will have depended, at least in part, on their physical factors. Unmeasured confounding can therefore not be excluded and these results must be treated with caution, as for any observational analysis.

The H4RT study aims to address these issues by randomly allocating patients to aim for high-volume HDF or receive standard of care (high-flux dialysis). Analysis will be by ITT, with no patients withdrawn from the analysis because they are unable to achieve high volumes of HDF. The primary analysis will also not censor patients at transplant, another criticism of earlier negative trials [8]. The efficient design of H4RT (relying on linkage to routinely collect healthcare data for outcomes) has kept the overall costs of this trial just above £2million and explores the opportunities to address clinically important questions in a relatively rare condition without commercial prospects. H4RT will report its findings in late 2025.

## Trial status

Recruitment commenced in November 2017 and is scheduled to continue to the end of September 2022. Follow-up is expected to continue to the end of May 2025. The current protocol is version 8, 3 May 2022.

## Supplementary Information

**Additional file 1:**

## Abbreviations

AE: Adverse event
AR: Adverse reaction
CFU: Colony-forming unit
CI: Chief investigator
CKD: Chronic kidney disease
CRF: Case report form
DMC: Data Monitoring Committee
DSI: Dialysis Symptoms Index
EQ-5D-5L: EuroQol 5-dimension 5-level
EU: Endotoxin units
GCP: Good Clinical Practice
HD: Haemodialysis
HDF: Haemodiafiltration
HES: Hospital Episode Statistics
HR: Hazard ratio
ICER: Incremental cost-effectiveness ratio
INMB: Incremental net monetary benefit
ISD: Information Services Division
ISRCTN: International Standard Randomised Controlled Trials Number
ITT: Intention to treat
MHRA: Medicines and Healthcare products Regulatory Agency
NHS R&D: National Health Service Research & Development
NIHR: National Institute for Health
NICE: National Institute for Health and Care Excellence
PEDW: Patient Episode Database Wales
PD: Peritoneal dialysis
PI: Principal investigator
PIS: Participant Information Sheet
PHE: Public Health England
QALY: Quality-adjusted life year
QoL: Health-related quality of life
QRI: Quintet Recruitment Intervention
RCT: Randomised control trial
REC: Research Ethics Committee
SAE: Serious adverse event
SAR: Serious adverse reaction
SUSAR: Suspected unexpected serious adverse reaction
TMG: Trial Management Group
TSC: Trial Steering Committee
UKRR: UK Renal Registry
UKHSA: UK Health Security Agency
