# Supplementary material for: The high-volume haemodiafiltration vs high-flux haemodialysis registry trial (H4RT): a multi-centre, unblinded, randomised, parallel-group, superiority study to compare the effectiveness and cost-effectiveness of high-volume haemodiafiltration and high-flux haemodialysis in people with kidney failure on maintenance dialysis using linkage to routine healthcare databases for outcomes
Source: Trials. 2022 Jun 27;23:532. doi: 10.1186/s13063-022-06357-y (PMC9235280; doi:10.1186/s13063-022-06357-y)
Supplement: Supplementary file 1 — Additional file 1: [file 13063_2022_6357_MOESM1_ESM.pdf]

The High-volume Haemodiafiltration

# vs High-flux Haemodialysis Registry Trial

# - H4RT -

Approvals and IDs	*Reference*
Protocol version	Version 8.0 3 May 2022
IRAS project ID	227067
REC Reference	17/SC/0391
Sponsor	North Bristol NHS Trust (R&I) ref 3859
ISRCTN	ISRCTN 10997319
Funder (NIHR HTA)	15/80/52
NIHR CRN Portfolio	34704

This protocol has regard for the HRA guidance and order of content

PROTOCOL VERSION NUMBER AND DATE

**Amendment No.**	**Protocol version no.**	**Date issued**	**Author(s) of changes**	**Details of changes made**
Pre-approval	0.1	3rd April 2017	Caskey	Recruitment end date corrected on page 8.
Pre-approval	0.2	21st May 2017	Caskey	Added Dr Albert Power to list of co-investigators.
Pre-approval	0.3	28th June 2017	Caskey	Qualitative research section amended in relation to verbal consent.
Pre-approval	0.4	4th July 2017	Caskey	Correction form in which withdrawal will be captured in section 7.8
	1.0	7th July 2017	Caskey	
Post-approval	2.0	21st Oct 2017	Caskey	(1) Removal of "Treatment with HDF for more than 3 months prior to inclusion in the trial or prior intolerance of HDF" as and exclusion criterion. (2) Option to follow up initial information with face-to-face visit, not just telephone.
Post approval	3.0	11th July 2018	Caskey	Addition of an analysis to look for an interaction effect according to baseline HDF experience (section 9.4)
Post approval	4.0	3rd October 2019	Caskey	(1) Health evaluation to include a secondary perspective of NHS and residential care, to include respite and permanent care in a nursing home, residential home or hospice. (2) The SAE reporting process has been revised. It is now clearer that local research teams need to check for adverse events monthly and establish whether or not they are unexpected

				SAEs, SARs or SUSARs. If they are they need expedited reporting to the CI and Sponsor. If not they need locally logged, but will be captured through linkage with hospital statistics. (3) The patient information sheet has been updated to include recommendations from the patient advisory group.
Post approval	5.0	24 June 2020	Caskey	H4RT patient recruitment poster for waiting areas of dialysis units to signpost patients who are interested in taking part in the trial to a local contact who can provide further information.
Post approval	6.0	15 July 2020	Caskey	Due to COVID-19, throughout the protocol: Face to face appointments must comply with local polices based on national guidance on social distancing and PPE; If clinically appropriate, patients can be approached by telephone/video call to minimise contact with the patient.
Post approval	7.0	2 September 2021	Caskey	1) The approval of NIHR contract variation following delays due to Covid 19, the following dates have been updated: recruitment extension till 30/09/2022; follow till 31/05/2025 and study end date till 30/09/2025.<br><br>2) In order to improve response rates for the 6 monthly questionnaires a) The patient questionnaire v4.0 has been shortened b) A covering letter signed by the local site principal investigator and research nurse will be sent with the questionnaires to patients. The protocol

				has been updated to reflect this change (Section 10).<br><br>3) The statistics and data analysis (Section 9) has been updated to reflect recruitment end date, length of follow-up and COVID-19 sensitivity analysis.
Post approval	8.0	3 May 2022	Caskey	1) The Bristol Randomised Trials Collaboration (BRTC) Clinical Trials unit is Bristol Trials Centre (BTC) with effect from 16/12/2021. All references to BRTC have been replaced with BTC.<br><br>2) Name change of National Institute for Health Research to National Institute for Health and Care Research in April 2022 (page 9)<br><br>3) Addition of 'Blood stream infections' to Table 2. (36-37)<br><br>4) Addition of details relating to adherence to protocol, plans to promote participant retention and complete follow-up, data management and data flow within the study (page 38-39)<br><br>5) Adverse events screening timelines and making clear that these are directed by the DMC (page 47).<br><br>6) Update of the Statistical analysis plan to take into account sensitivity analysis, subgroup analysis and interim analysis as agreed by TMG for the protocol paper publication (page 52-53)<br><br>7) Procedure to account for missing data updated to account for amount of

				missing data ((>10%) with potential reasons will be explored (page 55).<br><br>8) The option to administer questionnaires by telephone by the CTU to get responses from the patient to the EQ5D questions (page 59).<br><br>9) Appendix 1 table updated to make contents clearer e.g. added column headings (page 71).

SIGNATURE PAGE

The undersigned confirm that the following protocol has been agreed and accepted and that the Chief Investigator agrees to conduct the trial in compliance with the approved protocol and will adhere to the principles outlined in the Medicines for Human Use (Clinical Trials) Regulations 2004 (SI 2004/1031), amended regulations (SI 2006/1928) and any subsequent amendments of the clinical trial regulations, GCP guidelines, the Sponsor’s SOPs, and other regulatory requirements as amended.

I agree to ensure that the confidential information contained in this document will not be used for any other purpose other than the evaluation or conduct of the clinical investigation without the prior written consent of the Sponsor

I also confirm that I will make the findings of the study publically available through publication or other dissemination tools without any unnecessary delay and that an honest accurate and transparent account of the study will be given; and that any discrepancies from the study as planned in this protocol will be explained.

For and on behalf of the Study Sponsor:

Signature: Date:

..../....../......

Name (please print):

....

Position:

....

Chief Investigator:

Signature: Date:

..../....../......

Name: (please print):

....

Signatures continued over page

Statistician:

Signature:

....

Name: (please print):

....

Position:

....

KEY TRIAL CONTACTS

Chief Investigator	Professor Fergus Caskey<br>University of Bristol<br>Population health Sciences, Bristol Medical School<br>Canynge Hall, 39 Whatley Road<br>Bristol BS8 2PS<br>Email: fergus.caskey@bristol.ac.uk
Trial Manager	Dr Sunita Procter<br>University of Bristol<br>Population health Sciences, Bristol Medical School<br>Canynge Hall, 39 Whatley Road<br>Bristol BS8 2PS<br>Email: h4rt-study@bristol.ac.uk
Lead Research Nurse	Ms Karen Alloway<br>Clinical Research Centre<br>Southmead Hospital<br>Westbury on-Trym<br>Bristol<br>BS10 5NB<br>Email : Karen.Alloway@nbt.nhs.uk
Sponsor	Research & Innovation<br>North Bristol NHS Trust<br>Floor 3 Learning & Research Building<br>Southmead Hospital<br>Westbury-on-Trym<br>Bristol, BS10 5NB<br>Telephone: 0117 414 9330<br>Email: researchsponsor@nbt.nhs.uk

Funder	NIHR Health Technology Assessment Programme<br>National Institute for Health and Care Research<br>Evaluation, Trials and Studies Coordinating Centre<br>University of Southampton<br>Alpha House<br>Enterprise Road<br>Southampton, SO16 7NS<br>Email: netsmonitoring@nihr.ac.uk
Clinical Trials Unit	Bristol Trials Centre (BTC)<br>Population Health Sciences, Bristol Medical School<br>University of Bristol<br>1-5 Whiteladies Road<br>Bristol, BS8 1NU<br>Telephone: +44 117 455 1230
Co-investigators	Dr J Athene Lane, Professor in Trials Research, University of Bristol<br>Professor Jenny Donovan OBE, Professor of Social Medicine, University of Bristol<br>Mr Bud Abbott, Patient and Public Involvement, North Bristol NHS Trust<br>Dr Leila Rooshenas, Lecturer in Qualitative Health Science, University of Bristol<br>Professor William Hollingworth, Professor of Health Economics, University of Bristol<br>Professor Yoav Ben-Shlomo, Professor of Clinical Epidemiology, University of Bristol<br>Professor David Collins Wheeler, Professor of Kidney Medicine and Honorary Consultant Nephrologist, University College London

	Professor Margaret May, Professor of Medical Statistics, University of Bristol<br><br>Dr Andrew Davenport, Consultant, Royal Free Hampstead NHS Trust<br><br>Dr Sandip Mitra, Consultant Nephrologist, Central Manchester University Hospitals NHS Foundation Trust<br><br>Dr Stephanie MacNeill, Senior Lecturer in Medical Statistics<br><br>Professor Ken Farrington, Consultant Nephrologist, East and North Hertfordshire NHS Trust<br><br>Dr Albert Power, Consultant Nephrologist, North Bristol NHS Trust
Lead Statistician	Dr Stephanie MacNeill, Senior Lecturer in Medical Statistics<br><br>Bristol Trials Centre (BTC)<br><br>Population health Sciences, Bristol Medical School<br><br>University of Bristol<br><br>1-5 Whiteladies Road<br><br>Bristol, BS8 1NU<br><br>Email: stephanie.macneill@bristol.ac.uk

TRIAL SUMMARY

Trial Title	The High-volume Haemodiafiltration vs High-flux Haemodialysis Registry Trial	
Short title	H4RT	
Trial Design	A non-blinded randomised controlled trial comparing the clinical and cost-effectiveness of two dialysis methods – high-volume HDF and high-flux HD.	
Trial Participants	Adult patients on in-centre maintenance haemodialysis or haemodiafiltration for End Stage Kidney Disease (ESKD)	
Planned Sample Size	1550	
Treatment duration	32months (min) to 91 months (max)	
Follow up duration	32 months (min) to 91 months (max)	
Planned Trial Period	Recruitment between 01/11/2017 and 30/09/2022<br>Continue treatment and follow-up until 31/05/2025	
	Objectives	Outcome Measures
Primary	To determine the relative effectiveness of high-volume HDF compared with high-flux HD on non-cancer mortality and hospital admission due to a cardiovascular event or infection	Non-cancer mortality or hospital admission with a cardiovascular event or infection within 3 years
Secondary	• Mortality<br>• Morbidity<br>• Quality of life<br>• Indirect effects<br>• Cost-effectiveness	All-cause mortality, cardiovascular and infection related morbidity and mortality. Health-related quality of life (QoL), cost effectiveness and environmental impact.

FUNDING AND SUPPORT IN KIND

**Funder(s)**<br>(Names and contact details of ALL organisations providing funding and/or support in kind for this trial)	**Financial and non-financial support given**
NIHR Health Technology Assessment Programme<br>National Institute for Health and Care Research<br>Evaluation, Trials and Studies Coordinating Centre<br>University of Southampton<br>Alpha House<br>Enterprise Road<br>Southampton, SO16 7NS<br>Email: netsmonitoring@nihr.ac.uk	Grant funding
Bristol Trials Centre (BTC)<br>Population Health Sciences, Bristol Medical School<br>University of Bristol<br>1-5 Whiteladies Road<br>Bristol, BS8 1NU<br>Telephone: +44 117 455 1230	Methodological expertise
Research & Innovation<br>North Bristol NHS Trust<br>Floor 3 Learning & Research Building<br>Southmead Hospital<br>Westbury-on-Trym<br>Bristol, BS10 5NB<br>Telephone: 0117 414 9330<br>Email: researchsponsor@nbt.nhs	Sponsorship
UK Renal Registry<br>Floor 3 Learning & Research building<br>Southmead Hospital<br>Westbury-on-Trym	Partner

Bristol, BS10 5NB<br>Email: renalregistry@renalregistry.nhs.uk	

LIST OF CONTENTS

SIGNATURE PAGE 6

KEY TRIAL CONTACTS 8

TRIAL SUMMARY 11

FUNDING AND SUPPORT IN KIND 12

LIST OF CONTENTS 14

TRIAL FLOW CHART 19

19

1 BACKGROUND 20

2 RATIONALE 22

3 OBJECTIVES AND OUTCOME MEASURES/ENDPOINTS 24

3.1 Primary objective 24

3.2 Secondary objectives 24

3.3 Objectives of the internal pilot trial 24

3.4 Primary endpoint/outcome 24

3.5 Secondary endpoints/outcomes 24

4 TRIAL DESIGN 26

5 STUDY SETTING 27

6 ELIGIBILITY CRITERIA 28

6.1 Inclusion criteria 28

6.2 Exclusion criteria 28

7 TRIAL PROCEDURES......29
7.1 Recruitment, screening and consent......29
7.2 The randomisation scheme......30
7.3 Blinding......31
7.4 Baseline data......32
7.5 Trial assessments......33
–> Data collected through linkage or postal/online questionnaire
33
7.6 Long term follow-up assessments......35
Numbers in parentheses following diagnoses refer to the Healthcare Cost and Utilisation Project Clinical Classification System for mapping diagnoses onto ICD-10 www.hcup-us.ahrq.gov. Abbreviations: HES = Hospital Episode Statistics; ICD = International Classification of Diseases; ISD = Information Services Division; PEDW = Patient Episode Database for Wales; PHE=Public Health England; UKHSA = UK Health Security Agency; UKRR = UK Renal Registry......37
7.8 Withdrawal criteria......45

8 SAFETY......46
8.1 Definitions......46
8.2 Safety monitoring......46
8.3 Recording and reporting of unexpected SARs and SUSARs......47
8.4 Responsibilities......48

9 STATISTICS AND DATA ANALYSIS......49
9.1 Sample size calculation......49
9.2 Planned recruitment rate......50
9.3 Statistical analysis plan......52
9.4 Subgroup analyses......53
9.5 Adjusted analysis......53
9.6 Interim analysis......53
9.7 Subject population......55
9.8 Procedure(s) to account for missing or spurious data......55
9.9 Other statistical considerations......55
9.10 Economic evaluation......55

10 DATA HANDLING......59
10.1 Data collection tools and source document identification......59

10.2 Data handling and record keeping....59
10.3 Access to Data....62
10.4 Archiving....62

11 MONITORING, AUDIT AND INSPECTION....63

12 ETHICAL AND REGULATORY CONSIDERATIONS....64
12.1 Research Ethics Committee (REC) review and reports....64
12.2 Peer review....65
12.3 Public and Patient Involvement....65
12.4 Regulatory Compliance....66
12.5 Protocol compliance....66
12.6 Notification of Serious Breaches to GCP and/or the protocol....66
12.7 Data protection and patient confidentiality....66
12.8 Financial and other competing interests for the chief investigator, PIs at each site and committee members for the overall trial management....67
12.9 Indemnity....67
12.10 Amendments....68
12.11 Post trial care....68
12.12 Access to the final trial dataset....68

13 DISSEMINATION POLICY....69
13.1 Authorship eligibility guidelines and any intended use of professional writers....70

14 APPENDICES....71
14.1 Appendix 1 – Assessments and follow-up....71
14.2 Appendix 2 – Risk....72
14.3 Appendix 3 – Study management / responsibilities....73

15 REFERENCES....75

LIST OF ABBREVIATIONS

Define all unusual or 'technical' terms related to the trial. Add or delete as appropriate to your trial. Maintain alphabetical order for ease of reference.

AE	Adverse Event
AR	Adverse Reaction
CFU	Colony forming unit
CI	Chief Investigator
CKD	Chronic kidney disease
CR	Civil Registration
CRF	Case Report Form
DMC	Data Monitoring Committee
DSI	Dialysis Symptoms Index
EQ-5D-5L	EuroQol 5-dimension 5-level
ESKD	End-stage kidney disease
EU	Endotoxin units
GCP	Good Clinical Practice
HD	Haemodialysis
HDF	Haemodiafiltration
HES	Hospital Episode Statistics
HR	Hazard ratio
ICER	Incremental cost-effectiveness ratio
INMB	Incremental net monetary benefit
ISD	Information Services Division
ISF	Investigator Site File
ISRCTN	International Standard Randomised Controlled Trials Number
ITT	Intention to treat

MHRA	Medicines and Healthcare products Regulatory Agency
NHS R&D	National Health Service Research & Development
NICE	National Institute for Health and Care Excellence
PEDW	Patient Episode Database Wales
PD	Peritoneal dialysis
PI	Principal Investigator
PIS	Participant Information Sheet
QALY	Quality adjusted life year
QoL	Health-related quality of life
QRI	Quintet Recruitment Intervention
RCT	Randomised Control Trial
REC	Research Ethics Committee
SAE	Serious Adverse Event
SAR	Serious Adverse Reaction
SOP	Standard Operating Procedure
SUSAR	Suspected Unexpected Serious Adverse Reaction
TMG	Trial Management Group
TSC	Trial Steering Committee
TMF	Trial Master File

TRIAL FLOW CHART

The High-volume Haemodiafiltration vs High-flux Haemodialysis Registry Trial

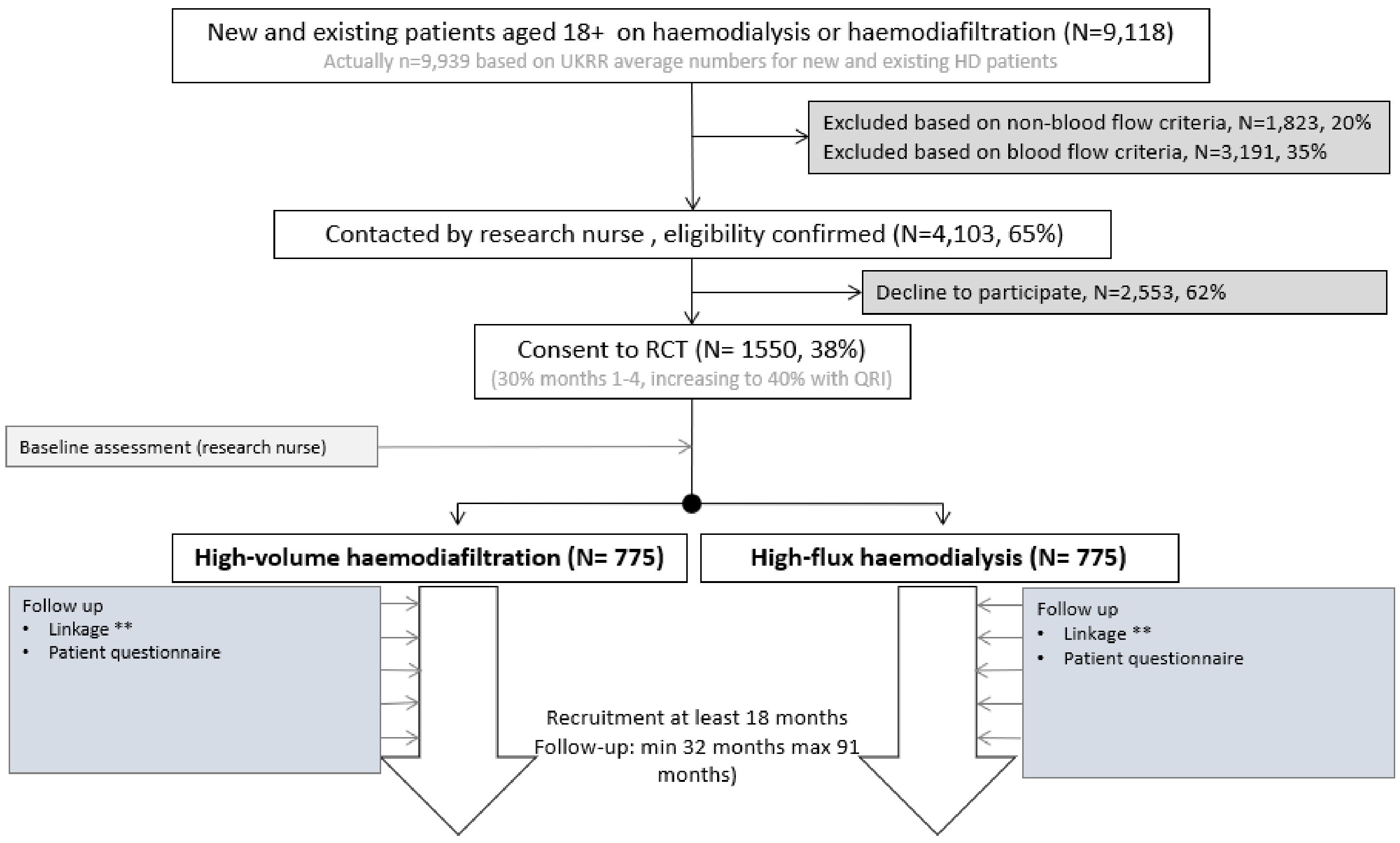

* Exclusion criteria:
Lack of capacity to consent; Clinician predicted life expectancy of less than 3 months; Started maintenance HD/ HDF within 4 weeks; Transition to living kidney donor transplant or home dialysis scheduled within 3 months; Dialysis less than thrice weekly; Unlikely to achieve sufficient blood flow rates with current vascular access

** to Hospital Episode Statistics, Civil Registration, Public Health England and the UK Renal Registry in England and the equivalent databases in Scotland.
QRI = Quintet Recruitment Intervention

STUDY PROTOCOL

The High-volume Haemodiafiltration vs High-flux Haemodialysis Registry Trial

1 BACKGROUND

End-stage kidney disease (ESKD) affects ~55,000 people in the UK, with ~7,000 newly affected people each year (1, 2). It ranks among the most severe of the chronic non-communicable diseases. The survival probability at one, three and five years is around 90, 70 and 50%, respectively (3). Morbidity is high, with dialysis patients in the UK admitted to hospital on average ~1.5-2.0 times per year and spending ~15 days in hospital per year (4). Quality of life on dialysis is also well below that of the general population (5). There is therefore an unmet and urgent need to improve ESKD patient treatment.

Renal replacement therapy (dialysis or transplantation) is necessary when approximately 90% of kidney function is lost. Currently ~90% of existing dialysis patients are on some form of haemodialysis (HD) or haemodiafiltration (HDF) (2). Although HD and HDF can be performed at home, the majority is performed in-centre.

HD relies on ‘diffusion’ – molecules at high concentrations in the blood pass across a membrane in an artificial kidney or dialyser to reach low concentrations in the dialysate fluid. At first, the pores in these membranes had to be small to avoid the loss of proteins and this meant that only small-sized toxic molecules could leave the blood. As technology advanced, these pores became larger and more complex/ asymmetrical, making it easier for larger toxic molecules to leave the blood whilst essential proteins are retained. These “high-flux” membranes are now recommended as standard practice in the UK (6). Even with these high-flux membranes, however, only the equivalent of 10-15% of toxin removal can be achieved within the timing/ frequency of a fairly standard dialysis prescription (i.e. 4 hours three times a week) (7).

HDF is similar to HD in that it uses diffusion to clean the blood (see above), but at the same time it uses ‘convection’ – a process that pulls fluid across the membrane, taking any dissolved solutes with it. When large volumes of fluids are pulled across the membrane (more than 23L per treatment session) it is considered ‘high-volume’ HDF. Adding convection achieves more efficient removal of middle-sized water soluble and even protein bound toxic molecules that cause cardiovascular damage, impaired immunity and other organ damage (8). This could explain why meta analyses of

existing randomised controlled trials indicate improved morbidity and mortality across a range of cardiovascular and infection-related outcomes in patients receiving high-volume HDF (9-12).

The current standard of care, high-flux HD, is water intensive: each treatment requires ~500L of mains water to generate ~120L of dialysate water (13). Given the exposure of the blood to such large quantities of water, it is important that chemicals and infections are kept below safe limits. For this reason, the water standard for high-flux HD is defined as “Ultrapure” (i.e. bacterial limits <0.1 CFU/mL & endotoxin limits <0.03 EU/mL). As high-volume HDF involves infusing an additional 20-25L of water back into the patient x3 per week, x52 weeks per year, the quality of water becomes even more crucial and has to meet “Sterile dialysate” standards (i.e. bacterial limits <10-6 CFU/mL & endotoxin limits <0.03 EU/mL). Technological developments over the past decade now make it possible to produce such water “on-line”, i.e. continuously in the renal unit, with filters built into dialysis machines that ensure sterile dialysate (14). This shifts responsibility for water quality to individual renal units and raises the importance of monitoring water quality if units are to provide a safe HDF service.

Box 1. Characteristics of high-flux HD and high-volume HDF

	**High-flux HD**	**High-volume HDF**
**Typical schedule**	~4 hours, x3 /week	~4 hours, x3 /week
**Diffusion**<br>• Water used (per treatment)	Yes<br>~120L	Yes<br>~120L
**Convection**<br>• Water used (per treatment)	No<br>0L	Yes<br>20-25L
**Total mains water used** (per treatment)	~500L	~600L
**Water purity**<br>• Bacterial limits<br>• Endotoxin limits	Ultra-pure<br><0.1 CFU/mL<br><0.03 EU/mL	Sterile<br><10-6 CFU/mL<br><0.03 EU/mL

The other concern about high-volume HDF is that the removed fluid may contain important solutes and proteins (such as albumin) that are not replaced in the sterile dialysate. This could have an adverse impact on a patient’s nutritional status (15).

2 RATIONALE

For patients with ESKD who are suitable for kidney transplantation the average waiting time in the UK is 2.8 years (16); minimising damage from ESKD during this time is likely to improve their long-term outcomes. For others, kidney transplantation is not an option and we need to optimise quality and quantity of life. Despite a lack of evidence of cost-effectiveness, ~15% of patients in the UK are currently receiving HDF, with wide centre variation and plans for further adoption (unpublished UKRR survey, Oct 2015). Before this technology diffuses more widely across the UK, a definitive trial is needed to determine whether HDF should be made available to all patients, certain sub-groups of patients, or none.

In addition to the impact of ESKD on the lives of affected individuals and their families outlined above, HD costs ~£25k per patient per annum. Treating the 25,000 people on high-flux HD costs around £500m of NHS spending each year (17), with a further £75m spent on hospital admissions and £50m on transport to and from dialysis (17). Half of patients now starting dialysis are 65 years or older and less likely to be fit for kidney transplantation and in the general population this group is predicted to increase by 60% (from 10.3m to 16.9m) by 2035 (18). While preventing ESKD in the first place should remain a priority, the optimal form of dialysis will remain highly relevant to the NHS.

Three meta-analyses have compared different forms of HDF with different forms of HD and drawn differing conclusions (10, 12, 19). One found no effect of HDF on all-cause mortality but included very old studies of HDF regimens very different from current practice (12); after removing these studies the relative risk of mortality became 0.82 (95% CI 0.72-0.93) (12). The other two found no significant effect on all-cause mortality overall (10, 19). Further, a post-hoc analysis of all the three recent major RCTs that included some patients on high-volume HDF (20-22) found significantly lower relative risk in those receiving the highest HDF volume (0.55, 0.34-0.84; 0.54, 0.31-0.93 and; 0.61, 0.38-0.98 in those achieving >25.4L, ~20.3L and >21.95L of convection per treatment, respectively) (10). The importance of HDF volume had not been appreciated when these trials were conceived and so varied widely from low volume to high volume. Detailed analysis of one of the RCTs has shown that most of the variation in HDF volume is explained by practice patterns, not patient characteristics (23). These practices that determine HDF volume are now well recognised and will be targeted in trial specific standard operating procedures designed to optimise the delivered HDF volume and minimise centre effects. QoL has only been reported in trials looking at older, lower volume HDF with no consistent evidence of benefit (12). There is also limited evidence on the cost-effectiveness of HDF. The main CONTRAST Study concluded that minor additional costs of HDF were not counterbalanced by a relevant QALY gain, but did not stratify by HDF volume in this analysis (24). A subsequent analysis from a single Canadian site that achieved high-volume HDF in the majority of its participants included

in the CONTRAST Study reported significantly higher QoL in patients on high-volume HDF and concluded that high-volume HDF was cost-effective in a Canadian setting (25). Numbers were small in this study (n=67 on HDF and n=63 on HD) and the comparator treatment was low-flux HD, rather than the current UK best practice of high-flux HD. The current UK Renal Association guideline states: “Haemodiafiltration would be the preferred mode of [dialysis] if it was shown in randomised controlled trials to provide better patient outcomes than high flux haemodialysis. Evidence level 2C” (6). Better quality evidence is therefore required.

3 OBJECTIVES AND OUTCOME MEASURES/ENDPOINTS

Aim: To establish the effectiveness and cost-effectiveness of high-volume HDF compared with high-flux HD in adult patients with ESKD on maintenance thrice weekly in-centre HD.

3.1 Primary objective

To determine the relative effectiveness of high-volume HDF compared with high-flux HD on non-cancer mortality and hospital admission due to a cardiovascular event or infection (primary outcome).

3.2 Secondary objectives

To determine the effect of high-volume HDF on the following secondary outcomes:

- Mortality: from all-causes as well as cause-specific
- Morbidity: hospital admissions related to cardiovascular events and infection events; reportable infections like MRSA and MSSA;
- Quality of life: generic, health utility, disease-specific and time to recover following dialysis.
- Indirect effects: laboratory indicators of inflammation, anaemia, bone mineral disorder management
- NHS costs and cost-effectiveness: Incremental cost-per QALY gained.

3.3 Objectives of the internal pilot trial

1. To rapidly identify barriers to recruitment using Quintet Recruitment Intervention methods and to address these to optimise informed consent and recruitment.
2. To establish the feasibility of recruiting to a fully-powered RCT of high-volume HDF compared with highflux HD amongst the participating centres.
3. To establish the generalisability of the sample recruited in relation to (i) the percentage of eligible patients agreeing to participate and (ii) the characteristics and outcomes of participating and nonparticipating patients.

3.4 Primary endpoint/outcome

A composite of first of non-cancer mortality or admission to hospital related to a cardiovascular event or infection (UKRR, Hospital Statistics & Civil Registration (CR)).

3.5 Secondary endpoints/outcomes

- All-cause mortality (UKRR & CR)

- Non-cancer mortality (CR)
- Cardiovascular - cause-specific hospitalisation & mortality (UKRR, Hospital Statistics (HES, PEDW, ISD & CR)
- Infection - cause-specific hospitalisation & mortality (UKRR, Hospital Statistics & CR) and reportable infections (MRSA & MSSA) (Public Health England)
- Health related quality of life – preference-based quality of life derived from EQ-5D-5L,, disease specific quality of life (Dialysis Symptom Index) and time to recover after each dialysis [1] (5)
- Indirect effects: routinely measured/ prescribed and recorded anaemia disorder management (haemoglobin levels and erythropoiesis stimulating agent dose), mineral bone disorder management (calcium, phosphate and PTH levels and phosphate binder dose) and nutritional status (albumin level) (UKRR)
- Cost-effectiveness from an NHS perspective and a second NHS & residential care perspective i.e. nursing home, residential home and hospice care whether for respite or permanent residence (UKRR, Hospital Statistics for in-patient and out-patient activity & targeted resource use questions for primary care, residential care, and medication usage)
- Impact on the environment, including locally purified water, manufactured saline and plastic consumables
- Water quality testing and breaches

[1] Answer to question “How long does it take you to recover from a dialysis session?”

4 TRIAL DESIGN

A non-blinded, randomised, parallel group, controlled trial comparing high-volume HDF (aiming for 21+L of substitution fluid adjusted to body surface area) against high-flux HD, randomised 1:1 and stratified by site, age (18-64 and 65+) and residual renal function (urine volume <100mL/day and 100+mL/day (26-28)). The primary analysis will be intent to treat using proportional hazards regression and adjusting for variables used to stratify the randomisation.

5 STUDY SETTING

This is a UK-wide, multi-centre trial recruiting patients from secondary care renal units either in a main dialysis setting or satellite dialysis unit.

6 ELIGIBILITY CRITERIA

6.1 Inclusion criteria

- Adult patients receiving in-centre, maintenance HD or HDF for ESKD;
- Dialysing at least three times a week in a main dialysis or satellite unit;
- Potential to achieve high-volume HDF.

6.2 Exclusion criteria

- Lack of capacity to consent;
- Clinician predicted prognosis of less than 3 months;
- Started maintenance HD or HDF within the preceding 4 weeks;
- Transition to living kidney donor transplant or home dialysis scheduled within next 3 months;
- Not suitable for high-volume HDF for other clinical reasons such as dialysis less than thrice weekly or unlikely to achieve sufficient blood flow rates with current vascular access, or prior intolerance of HDF.

7 TRIAL PROCEDURES

7.1 Recruitment, screening and consent

Dialysis is provided by renal units, either in a main dialysis unit (with a nephrologist on site) or in a satellite dialysis unit (without a nephrologist on site). For adult patients, there are 71 main dialysis units and ~164 satellite dialysis units (235 units in total). Eligible patients will be dialysing three times a week in one of these units and each unit will know exactly who these patients are and when they will next be attending, with never more than 3 days between attendances. To optimise the efficiency of recruitment, potentially eligible patients will be approached according to their regular dialysis shift, thus enabling 2-3 patients to be recruited in a half-day/ single visit to a dialysis unit. These arrangements will be individualised according to local circumstances at each site. A patient recruitment poster in waiting areas of dialysis units may be used to signpost patients who are interested in taking part in the trial to a local contact who can provide further information.

Due to the COVID-19 pandemic, throughout the protocol: Face to face appointments must comply with local polices based on national guidance on social distancing and PPE; If clinically appropriate, patients can be approached by telephone/video call to minimise contact with the patient.

Identification, screening and consent procedures will be undertaken by research staff and treating clinicians who will be trained and competent to participate according to the ethically approved protocol, principles of Good Clinical Practice (GCP) and Declaration of Helsinki. It will take place in several steps:

1. Nurses in dialysis units will provide a list of potentially eligible patients in their units
2. Eligibility will be confirmed by the patient’s treating clinician or the local principal investigator.
3. Standard letters will be sent out/ handed out to potentially eligible patients introducing the study and including a Patient Information Sheet.
4. Letters will be followed up with a phone call/ face-to-face visit from the research staff to offer further discussion about the study/a baseline visit at a scheduled dialysis attendance.
5. Potentially eligible patients will be approached according to their regular dialysis shift.
6. Permission will be sought for patients to be allowed to measure their urine volume prior to the recruitment visit, thus enabling randomisation to take place at that visit. (If a 24 hour urine volume is available from the 6 weeks prior to randomisation, this can be used/ does not need repeating.) Randomisation will take place once this information is available and the participant and their dialysis nurses informed.
7. Each participant will be asked to provide written informed consent to be randomised to high-volume HDF or high-flux HD, and followed up through their routine health records and postal

questionnaires. The Patient Information Sheet and the Consent Form will explain the need for long term follow up and linkage to other routine health databases.

8. Patients who are not willing to be randomised, but who would otherwise be eligible, will be asked to consent to other research (e.g. interviews to explore their views on the quality of information provided about the trial, and how they reached their decision about participation) and linkage to explore differences in characteristics and outcomes between participants and non-participants).

7.2 The randomisation scheme

Patients will be randomised on a 1:1 basis to the “high-volume HDF” or “high-flux HD” treatment arms stratified by site to ensure a balance in terms of local differences, age (18-64 years and ≥65 years) and residual renal function (urine volume <100 mL/day and ≥100 mL/day).

Randomisation will be done using the BTC Randomisation System, which provides a secure service to generate allocations. This is a validated system.

The system is available 24 hours a day with minimal downtime over several years. System data are backed up daily.

7.2.1 Method of implementing the allocation sequence

All patients who enter the study will be logged with the central trial office and given a unique Study Number. The research staff will retrieve the information necessary for randomisation from the clinical record, i.e. site, age 18-64 and 65+ and residual renal function (urine volume <100mL/day and 100+mL/day. Participants will then be randomly allocated 1:1 to the “high-volume HDF” or “high-flux HD” treatment arms.

Randomisation will utilise the existing remote automated computer randomisation application at the study administrative centre in the BTC, a fully registered UK CRC clinical trials unit in the University of Bristol. This randomisation application will be available both as a telephone-based system and as an internet based service.

The BTC Randomisation system provides for layered security with access granted to BTC Data Management staff to be able to monitor the system. The system fails over to a backup system in the event of a system problem. Randomisation data are routinely backed-up to tape. In addition these data are synchronised to a secondary system every 15 minutes. This secondary system can act as a fall-back, in the event of a failure of the primary system.

The system logs all actions and can be configured to send an email on randomisation, with allocation and any other variable used in the process.

7.3 Blinding

Due to the nature of the intervention, participants and those administering the intervention will not be blinded to group allocation. The statistician performing the analysis will be blinded to the treatment allocation.

7.4 Baseline data

Clinical and patient reported data will be collected by research staff at baseline (following consent and prior to randomisation; see Table 1). Validated questionnaires will be used for patient reported outcomes (see section 3.5).

Table 1 Summary of baseline data collection for the randomised controlled trial

Demographics/social	Age, sex, ethnicity, marital status, education level, smoking history.
Clinical	Primary renal disease, date first seen by nephrologist, RRT treatment history, co-morbidities, dietary restrictions, prescribed medication (including erythropoiesis stimulating agents and phosphate binders), 24-hour urine volume (within the 6 weeks preceding randomisation).
Resource use	Day case and inpatient hospital admissions (including surgical procedures performed), nursing home/residential home days/hospice days, other hospital outpatient services and primary care & community services in the last 6 months.
Laboratory	Creatinine, urea, Kt/V, urea reduction ratio, albumin, haemoglobin, haematocrit, mean corpuscular volume, sodium, potassium, bicarbonate, corrected calcium, phosphate, c-reactive protein, intact parathyroid hormone, total cholesterol. (From the date of the study visit or the closest date prior to the study visit.)
Physical assessment	Height, weight, blood pressure, heart rate.
Patient reported	EQ-5D-5L, DSI, and time to recovery (5).

7.5 Trial assessments

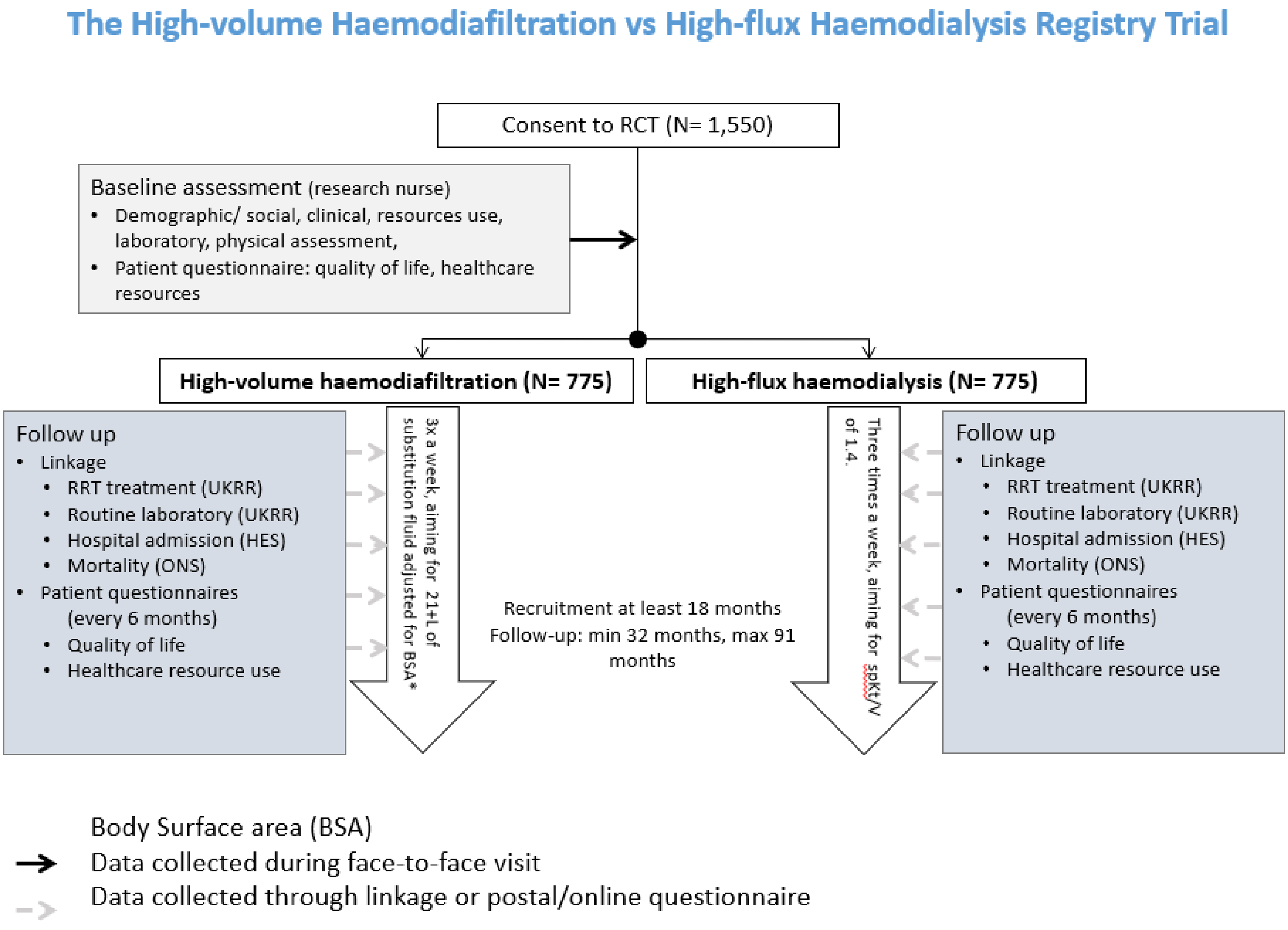

Figure 1 Overview of trial assessments

7.5.1 Intervention

The Intervention is in-centre, high-volume HDF which is usually delivered for ~4 hours three times a week. Each treatment will aim for 21+L of substitution fluid adjusted to body surface area[2] (i.e. 23+L of convection and 21+L of substitution, as ~2L of fluid normally needs to be removed and not replaced at any standard HD session to avoid people with kidney failure retaining fluid). This requires sterile water (bacterial limit <10-6 colony forming units (CFU) per

[2] With adjustment for pre-dilution 29. Tattersall JE, Ward RA, group E. Online haemodiafiltration: definition, dose quantification and safety revisited. Nephrol Dial Transplant. 2013;28(3):542-50. (if used) and amputation 30. Colangelo PM, Welch DW, Rich DS, Jeffrey LP. Two methods for estimating body surface area in adult amputees. American journal of hospital pharmacy. 1984;41(12):2650-5. (if present).

mL; endotoxin limit <0.03 Endotoxin Units (EU) per mL). A dialysis adequacy of spKt/V of 1.4 (see below) will be targeted.

It has been shown to be possible to achieve these convection volumes in 87% and 84% of people on dialysis via an arteriovenous (AV) fistula and graft, respectively; it is more difficult with a plastic neck line (14). If the dialysis time is not to be increased, which would introduce another confounding factor and reduce the appeal of the HDF to patients, the main factor in achieving high volume convection is the blood flow rate – the number of mL of blood that can be taken from the patient via their AV fistula or graft and passed through the dialyser in a minute (14). A standard operating procedure targeting for example dialysis needle gauge and blood pump speed will be developed by the investigators to assist dialysis nurses in optimising blood flow rates and therefore attaining the target convection volume.

7.5.2 Comparator

The Comparator is in-centre, high-flux HD (usual care), which is usually delivered for ~4 hours three times a week. It will require ultrapure water (bacterial limit <0.1 CFU per mL; endotoxin limit <0.03 EU per mL) and aim for the same dialysis adequacy of spKt/V of 1.4 as in the HDF arm. As mentioned above, patients need on average 2L of fluid removal on each HD treatment to avoid chronic fluid retention, but this is just removal and there is no replacement/ substitution.

The proposed water quality standard for the HD arm is higher than is currently required by the UK Renal Association Clinical Practice Guideline, which sets limits of <100 CFU per mL and <0.25 EU per mL (6). However, all 33 renal units responding to our survey reported working to the ultra-pure water standard, perhaps because most are using high-flux dialysers, which require higher quality water than low-flux dialysers due to the larger pores in the membrane. We feel it is important to specify this requirement for ultra-pure water so as to avoid any observed difference in outcomes being due to an inappropriate water quality standard being delivered to some patients in the HD arm.

7.5.3 Commonality between intervention and comparator

Dialysis dose - Clinical practice guidelines have set standards for small-sized toxin removal on dialysis – single pool Kt/V (spKt/V) and urea reduction ratio (6). There are no standards for middle- or large-sized toxin removal. In this trial, the small-sized toxin dialysis dose standard for both arms is slightly higher than the minimum spKt/V recommended in the NICE-approved UK Renal Association Clinical Practice Guidelines, 1.3 (6), as aiming for high volumes of convection in the HDF arm may increase small - sized toxin clearance (i.e. spKt/V). We would like to standardise this between the two arms even though the HEMO Study found no survival benefit from delivering an spKt/V of 1.5 vs 1.3 (31).

Use of high-flux dialysers - Dialysis relies on the blood passing through a filter (dialyser) which keeps the blood on one side of a semi-permeable membrane and ultra-pure fluid (dialysate) on the other. Toxins diffuse down the concentration gradient from the high concentration in the blood, through the pores in the membrane, into the low concentration in the dialysate and thus out of the body.

Low-flux dialysers have small pores which only allow small-sized toxins to leave the blood, whereas high-flux dialysers have larger pores and therefore allow more middle-sized toxins to leave the blood. This has the potential to improve toxin removal from the blood. Although the UK Renal Association Guidelines do not specify whether low- or high-flux dialysers should be used (6), the European Renal Best Practice Guidelines have been updated following the Membrane Permeability Outcome Study (32) to recommend high-flux dialysers in all patients (33). All but 3 of the 33 renal units responding to our 2015 survey were routinely using high-flux dialysers. Having low-flux dialysers in the comparator group has been a criticism of prior RCTs of HDF vs HD (11).

Super high-flux and middle cut-off dialysers that are not suitable for haemodiafiltration (e.g. Baxter's Theranova dialyser) cannot be used for patients allocated to high-flux HD.

7.5.4 Difference between intervention and comparator

In both HDF and HD, toxins are removed by diffusion. The difference between the two treatments is that HDF also involves (i) convection to remove 23+L of toxin-containing fluid and (ii) substitution/ replacement of that volume with 21+L of fluid. As this fluid is being given directly into the patient's blood stream, it needs to be of a high degree of purity than is required for high-flux HD – sterile rather than ultra-pure (see Box 1).

For the patient, the treatments will appear very similar. The fluid removal and substitution/replacement occurs "within" the dialysis machine. Patients in both arms will need to come in to their dialysis unit for treatment three times a week for ~4 hours each time. Access to the blood for most patients is likely to be an AV fistula or graft so that sufficient blood flows can be achieved (i.e. not a plastic neck line, which is used in ~15% of patients). These AV fistulae and grafts will be needled in the same way for both groups for the blood to circulate out through the dialysis machine, though the needle gauge and blood pump speeds may need to be increased slightly in the HDF group to achieve the necessary convection volume.

7.6 Long term follow-up assessments

Follow up will continue for a minimum of 32 months and a maximum of 91 months. It will be undertaken through a combination of 6-monthly patient questionnaires and linkage to routine healthcare databases

such as the UK Renal Registry, Hospital Statistics (for England/ Wales/ Scotland and/or Northern Ireland), Civil Registration (**Table 2**). Only data that are collected as part of routine care will be collected. Paper and electronic (web portal) options will be offered to patients for patient questionnaire completion.

Adherence to the protocol will be monitored through UK Renal Registry treatment modality returns and contact with dialysis units throughout the follow up. As the UK Renal Registry follows all patients on RRT in the UK, patients should not be lost to follow-up unless they move to another country.

Table 2 Summary of follow-up data collection

Numbers in parentheses following diagnoses refer to the Healthcare Cost and Utilisation Project Clinical Classification System for mapping diagnoses onto ICD-10 www.hcup-us.ahrq.gov.

	Data items	Source
Routine laboratory data	Creatinine, urea, Kt/V, urea reduction ratio, albumin, haemoglobin, haematocrit, mean corpuscular volume, sodium, potassium, bicarbonate, corrected calcium, phosphate, c-reactive protein, intact parathyroid hormone, total cholesterol.	UKRR
Cardiovascular and infections hospital admission data	*Cardiovascular*: Nonspecific chest pain (102), Congestive heart failure; non-hypertensive (108), Coronary atherosclerosis (101), Other circulatory disease (117), Acute myocardial infarction (100), Peripheral and visceral atherosclerosis (114), Chronic ulcer of skin (199), Gangrene (248), Aortic; peripheral; and visceral arterial disease (115), Transient cerebral ischemia (112), Cardiac arrest and ventricular fibrillation (107), Pulmonary heart disease (103), Other and ill-defined cerebrovascular disease (111), Acute cerebrovascular disease (109).<br><br>*Infection*: Pneumonia (122), Septicemia (except in labour) (2), Pleurisy; pneumothorax; pulmonary collapse (130), Aortic and peripheral arterial emboli (116), Tuberculosis (1), Mycoses (4), HIV infection (5), Encephalitis (77), Meningitis (76),	Hospital Statistics (HES, PEDW, ISD)

	Shock (249), Skin and subcutaneous tissue infection (197), Fever of unknown origin (246), Infective arthritis and osteomyelitis (201), Bacterial infection; unspecified site (3), Other inflammatory condition of skin (198), Other infections; including parasitic (8), Influenza (123), Urinary tract infections (159), Genitourinary symptoms and ill-defined conditions (163)	
Mortality data	Non-cancer mortality (i.e. all causes of death excluding chapter II causes in ICD-10).	NHS Spine tracing, UKRR, Hospital Statistics, Civil Registration
Patient reported outcomes	EQ-5D-5L, DSI and Time to recovery (following dialysis) (5).	Patient questionnaire administered 6 monthly
RRT use	Frequency, machine, dialyzer, dialysis times and consumables used.	*Annual census (extracted from the renal IT system).*
Other hospital admissions	Day case and inpatient hospital admissions (including surgical procedures performed),	Hospital Statistics (HES, PEDW, ISD)
Patient reported healthcare use	Nursing home/residential home days/hospice days, and primary care, community services and medication usage in the last 6 months.	Patient questionnaire administered 6 monthly
Blood stream infections	Methicillin resistant staphyloccus aureus, Methicillin sensitive staphyloccus aureus, Clostridium difficile, and Escherichia coli, as report by mandate to PHE/ the UKHSA	Reported to PHE/ UKHSA and shared with UKRR

Numbers in parentheses following diagnoses refer to the Healthcare Cost and Utilisation Project Clinical Classification System for mapping diagnoses onto ICD-10 www.hcup-us.ahrq.gov.
Abbreviations: HES = Hospital Episode Statistics; ICD = International Classification of Diseases; ISD =

Information Services Division; PEDW = Patient Episode Database for Wales; PHE=Public Health England; UKHSA = UK Health Security Agency; UKRR = UK Renal Registry

Adherence to the protocol will be monitored through monthly reports from sites during recruitment reducing to three-monthly during follow-up. For the purposes of reporting only the last recorded dialysis session of each month will be examined. The log will include receipt of the allocated modality, attainment of high-volume HDF (if applicable), and water quality testing. Deviation from the protocol has been defined as (a) being off their allocated treatment on the last dialysis session of the month for two consecutive months and (for those allocated to high-volume HDF) (b) no longer aiming for high-volume HDF. This definition recognises that some patients need time to adjust their dialysis prescription and achieve their target substitution volume; indeed some, despite all best efforts, may never achieve it. It also recognises that patients may have short periods off their allocated treatment due to admission to hospital or travel with dialysis in another centre.

As the UKRR follows all patients on kidney replacement therapy in the UK, patients should not be lost to follow-up unless they move to another country or opt out of data linkage after being randomised.

Plans to promote participant retention and complete follow-up

As the primary outcome relies on data linkage with national routine healthcare databases, participants will only be lost to follow up if they opt out of data linkage after being randomised. Patients who discontinue allocated treatment can choose to continue to receive questionnaires and allow data linkage. The minimum number of identifiers will be used to link with routine data sources, as agreed with those organisations and set out in the participant information sheet. For patient-reported outcomes, up to three reminders are sent.

Data management

Baseline data will be collected on paper by the local research nurses. Forms will be copied, with originals stored locally and copies transferred securely in tamper-proof envelopes to the trials unit for entry into the database. Staff at the trials unit will mail or email out the follow up patient questionnaires, according to the participant's stated preference, and enter the returned questionnaires into the database. Study data will be collected and managed using REDCap hosted at the University of Bristol. The database incorporates data entry and validation rules to reduce data entry errors, and management functions to facilitate auditing and data quality assurance.

Identifiable information, as agreed with partner organisations and set out in the participant information sheet, will be used to link this primary dataset with existing routine healthcare data bases for follow up. The flow of data – identifiable and pseudonymized – is summarized in **Figure 2**. The database system will protect patient information in line with the data protection legislation and any specific requirements of the partner organisations. Trial staff will ensure that participants' anonymity is maintained through

protective and secure handling and storage of patient information. The chief investigator (CI) will act as custodian of the full dataset.

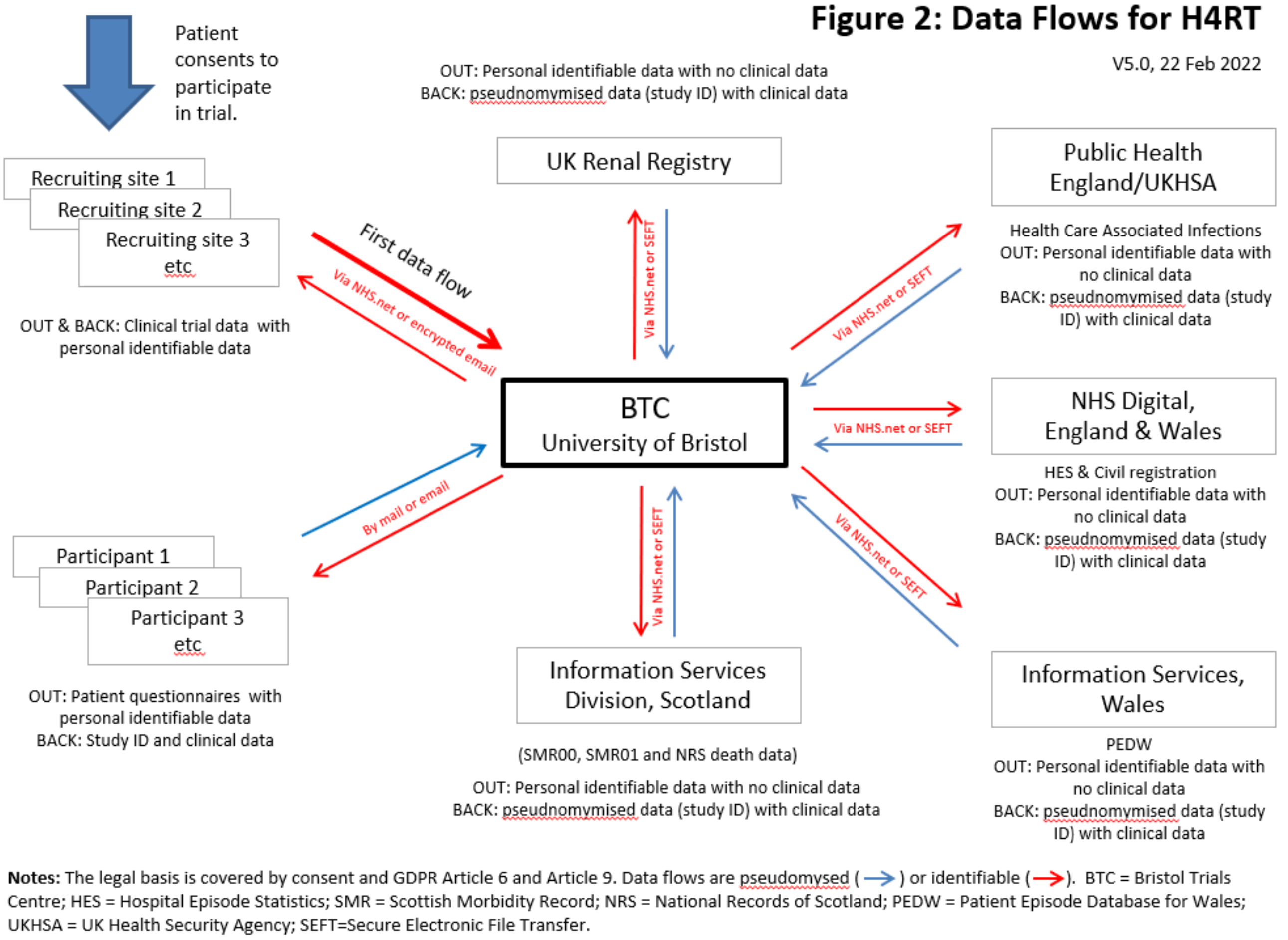

Figure 2: Data Flows for H4RT

7.7 Nested studies: QuinteT recruitment intervention

The QuinteT Recruitment Intervention (QRI) (34) will be integrated throughout the H4RT recruitment period, with the aim of optimising recruitment and informed consent. Recruitment may be challenging if clinicians, nurses, or patients have strong preferences for HD or HDF. There may also be unforeseen logistical challenges to randomising patients to a treatment (HDF) that is not yet fully integrated into clinical practice in some centres. The QRI assimilates investigation of generic and centre-specific recruitment challenges, with a combination of pre-emptive and responsive feedback/training.

The QRI will attempt to identify sources of recruitment difficulties as they occur and implement generic or bespoke strategies to address these. Recruitment processes will be investigated in depth across a

small number of clinical centres (i.e. 3 or 4) in the early phases of recruitment, with reviews of other centres as they open and recruitment proceeds. There will be an attempt to ensure these initial centres are as diverse as possible (e.g. in terms of size, current use of HDF, etc.). Lessons learnt from the QRI will subsequently be applied to other centres, combined with continued investigation of recruitment challenges.

The QRI uses novel qualitative and mixed-method approaches pioneered during the NIHR HTA-funded ProtecT (Prostate testing for cancer and Treatment) study (35). These methods have since been refined and applied to several other RCTs in different clinical contexts, all of which have led to insights about recruitment issues (36-38) and the development of recruitment strategies (34, 39). The QRI will proceed in two iterative phases: sources of recruitment difficulties are rapidly investigated in Phase I, informing a mix of generic and tailored interventions to improve recruitment in phase II.

7.7.1 PHASE I: understanding recruitment

Phase I aims to understand the recruitment process and how this operates in clinical centres. A multi-faceted, flexible approach will be used to investigate site-specific or wider recruitment obstacles. These will comprise one or more of the following methods of data collection:

a) In-depth interviews

Semi-structured interviews will be undertaken with three groups: (i) members of the Trial Management Group (TMG), (ii) clinicians or researchers who are involved in trial recruitment (‘recruiters’), and (iii) eligible patients who have been approached to take part in the trial. Interviews with members of the TMG and recruiters will explore their perspectives on the RCT and experiences of recruitment. Key topics explored will include: perspectives on the trial design; views about the evidence on which the trial is based; perceptions of equipoise; perceived barriers and facilitators to recruitment; integration of the trial in clinical centres, and any difficulties in implementing the trial protocol. Interviews with patients will explore views on the presentation of study information, understandings of trial processes (e.g. randomisation), and reasons underlying decisions to accept or decline the trial. Patients will be purposefully selected, to build a sample of maximum variation based on the centre/clinic they attend, their final decision about trial participation (i.e. accept or decline), and any other clinical (or non-clinical) characteristics that are deemed to potentially have a bearing on their decisions about trial participation. Some of these characteristics will likely emerge from interviews with clinical professionals. Numbers of interviews for each group of informants will be guided by the concept of ‘data saturation’ – the need to continue sampling until no new themes emerge.
All interviews will be audio recorded on an encrypted device, and take place at a mutually convenient location, in a suitably private and quiet setting. All participants will be offered the option to conduct the interview over the telephone. The University of Bristol’s ‘lone researcher’

safety policies will be upheld for any interviews taking place in non-public settings (e.g. participants' homes).

b) Audio-recording recruitment discussions

Scheduled appointments during which the H4RT is discussed with patients, including telephone conversations, will be audio-recorded on an encrypted device (and potentially observed) with written informed consent. These recordings/observations will be used to explore information provision, recruitment techniques, management of patient treatment preferences, and reasons underlying trial-participation decisions. Recording/observing appointments will also enable comparison of reported and actual recruitment practices for recruiters have also participated in interviews. Recordings will be collected by trial staff across the clinical centres, and transferred to and from the University of Bristol through University of Bristol-approved secure data transfer facilities or encrypted flash drives that adhere to NHS Trust policies.

c) Mapping of eligibility and recruitment pathways

Detailed eligibility and recruitment pathways will be compiled for clinical centres, noting the point at which patients receive information about the trial, which members of the clinical team they meet, and the timing and frequency of appointments. Recruitment pathways will be compared with details specified in the trial protocol and pathways from other centres to identify practices that are potentially more or less efficient. The QRI researcher will also work closely with the clinical trials unit (CTU) to compose detailed logs of potential participants as they proceed through screening and eligibility phases. This will help to identify points at which patients do not continue with recruitment to the RCT, thus indicating aspects of the recruitment process that may warrant further investigation and/or intervention.

Logs of eligible and recruited patients will be assembled using simple flow charts and counts to display numbers and percentages of patients at each stage of the eligibility and recruitment processes. These figures will be compared across centres, and considered in relation to estimates specified in the grant application/study protocol.

d) Observation of TMG and investigator meetings

The QRI researcher will regularly observe TMG meetings to gain an overview of trial conduct and overarching challenges (logistical issues, etc.). These meetings may be audio-recorded, subject to written informed consent.

7.7.2 PHASE 2: Development and implementation of recruitment intervention strategies

If recruitment difficulties are evident across the study or in particular centres, the QRI team will work closely with the TMG/CI to formulate a 'plan of action' that intends to improve recruitment and information provision. The components of this plan will be grounded in the findings from

phase 1, and may include generic, centre-specific, or individually-targeted interventions. Generic forms may include ‘tips’ documents that provide suggestions on how to explain trial design and processes, or changes to trial documentation and trial processes. Supportive feedback is likely to be a core component of the plan of action, with the exact nature and timing of feedback dependent on the issues that arise. Centre-specific feedback may cover institutional barriers, while multi-centre group feedback sessions may address widespread challenges that would benefit from discussion. All group feedback sessions will be aided by displaying anonymised data extracts from interviews and audio-recorded consultations. Individual confidential feedback will also be offered – particularly where recruiters experience specific difficulties, or where there is a need to discuss potentially sensitive issues. Investigator meetings/teleconferences and site visits from the CI/TMG members may also be employed to discuss technical or clinical challenges related to the trial (e.g. discomfort surrounding eligibility criteria).

7.7.3. Iterative nature of Phase I/Phase II

The QRI has been presented as two distinct phases for clarity, although in reality these are likely to overlap. For instance, new avenues of enquiry will emerge throughout the conduct of the QRI (e.g. in feedback meetings), and rigorous monitoring of screening logs before/after interventions may indicate a need for further investigations (phase I) or intervention (phase II).

7.7.4. Evaluating the ‘plan of action’

The impact of QRI interventions implemented in phase 2 will be evaluated through mixed approaches, including ‘before/after’ comparisons (number of recruited patients, eligible patients identified, patients accepting allocation) and investigation of changes in recruiter practice (through continued analysis of audio-recorded appointments). Semi-structured interviews will be conducted with recruiting staff and TMG members to explore their views on QRI interventions and suggestions for areas that would benefit from continued QRI input.

a) Quantitative evaluation

Information about recruitment plans and targets specified in the trial documentation (protocols/funding application) will be recorded prior to the start of recruitment. This will include:

- The target recruitment figures (ideally for each centre, per month). If a target recruitment line has been provided as a figure (i.e. image), the raw data informing this line should be requested from the TMG or CTU overseeing the study. Where possible, the rationale behind these targets should be explored and recorded.
- The planned period of recruitment

- The planned number of centres

Recruitment data will be regularly collected (e.g. at least monthly) throughout the recruitment period. As a minimum, this will include the number of patients randomised per centre, per month. Ideally, the number of patients screened, eligible, and approached will also be routinely collected per centre, per month.

The timing of interventions stemming from the QRI should be recorded in the form of day/month/year, with a brief description of the activity. All activities should be recorded, including (but not restricted to):

- Feedback of ‘phase 1’ findings to the CI and/or TMG, including details of the agreed ‘plan of action’ (and any subsequent plans for intervention).
- ‘Global interventions’ (not specific to any particular centre – e.g. ‘tips and guidance’ documents, changes to PILs, early discussion of findings with the chief investigator)
- ‘Centre-specific interventions’ (e.g. individual or group feedback within a centre).

b) Qualitative evaluation:

Reflective interviews will be conducted with key informants once the collaboration with the QuinteT team is drawing to an end. Key informants will constitute any individuals who have been exposed to QRI interventions or had a role in delivering the QRI. This will likely include the CI, trial manager/coordinator, and recruiters who have received feedback/training. Interviews will take place face to face or over the phone, and be informed by a flexible topic guide informed by previous work in this area (40). Ideally, interviews will be conducted by an independent member of the QuinteT team who has had no prior direct involvement in the RCT.

7.7.5. Consent processes for the QuinteT Recruitment Intervention

a) Health care professional consent

Recruiting staff and TMG member consent will be obtained through a ‘master’ consent form that covers all aspects of the QRI. The consent form will set out individual clauses, with the option to select ‘Yes’ or ‘No’ for each research activity accordingly. Research staff or the QuinteT researcher will obtain written consent from all staff. This will be a one-off process to cover consent for all future recordings of appointments, interviews, and observations of TMG/investigator meetings throughout the study.

b) Patient consent

***Audio recording/observing recruitment appointments*:**

Patients will be sent a copy of the QRI information sheet in the post, alongside the main Patient

Information Sheet about the RCT or given both PISs in an initial face to face discussion. Patients will be provided with sufficient time to read the information, ask any questions, and consider their participation in the QRI study.

A two-step consent process will be adopted for audio-recording initial telephone discussions about potential participation in the H4RT study. Research staff will check to make sure the patient has read and understood the QRI information sheet sent in the post, prior to the telephone discussion. Patients will then be asked to provide verbal consent for the telephone discussion to be audio-recorded. Patients who provide verbal consent will subsequently be asked to provide written informed consent for the audio-recording process at their next face-to-face appointment. Future discussions about potential H4RT participation will be audio-recorded subject to receiving this written consent; if patients choose not to provide written consent, the recording made from their initial telephone discussion will be deleted, and no further recordings made. Patients approached face to face will be asked to consent or decline participation in the QRI at the start of the second face to face discussion.

Interviews:

Patients will have received information about the interview processes in advance, in the QRI information sheet given or sent in the post. The research staff will reinforce this by explaining the interview process when they discuss the H4RT study processes with patients over the phone and in the face-to-face recruitment appointments. Written consent for the interviews will then be sought during a face-to-face appointment, once the patient has had sufficient time and opportunity to consider their participation in this part of the research. The QRI consent form will include a clause that asks patients if they would be willing to be take part in a future research interview ('Yes' or 'No'). Patients who select 'Yes' may then be approached by the qualitative researcher.

7.7.6. Analysis of QuinteT Recruitment Intervention data

Full or targeted sections of interviews and audio-recorded appointments will be transcribed verbatim by an approved transcription service/transcriber that has signed the necessary confidentiality agreements with the University of Bristol. All transcripts will be edited to ensure anonymity of respondent. Data will be managed using NVivo software and stored on encrypted drives at the University of Bristol, in line with the university's data storage policies.

Interview data will be analysed thematically using constant comparative approaches derived from Grounded Theory methodology (41). Analysis will be led by the member of the QuinteT team employed to deliver the QRI, with a sample of transcripts from each of set of stakeholder

interviews double coded by a second member of the team. An initial coding frame will be agreed for each set of interviews and reviewed as it evolves through further data collection and analysis. There will an attempt to search for negative cases in relation to themes, and emerging findings will be regularly discussed in team meetings. Evolving descriptive accounts of emerging findings will be prepared throughout the analytical process.

Audio-recorded recruitment consultations and follow up discussions will be subjected to content, thematic, and novel analytical approaches, including targeted conversation analysis (42) and appointment timing (the ‘Q-Qat method’) (43). There will also be a focus on aspects of information provision that are unclear, disrupted, or potentially detrimental to recruitment and/or adherence. Thematic approaches, and techniques to maintain rigour, will be similar to those described above (for interviews) (43). (43)

Notes from observations of appointments and TMG/investigator meetings will be recorded in a detailed log. Key issues/themes from these notes will be considered alongside emerging findings from interviews and audio-recorded appointments.

Findings from the above sources will be brought together and reported in descriptive accounts and summary reports, and presented to the CI and TMG. The content of these reports will focus on key recruitment issues identified, and potential solutions to address these.

7.8 Withdrawal criteria

The physician responsible for a patient retains the right to advise withdraw of a patient from a trial for appropriate medical reasons, be they individual adverse events or new information gained about a treatment. Participants can withdraw from (a) complying with the allocated trial treatment or (b) providing data to the trial, at any time for any reason without affecting their usual care. In both cases all ethically appropriate efforts will be made to report the reason for withdrawal as thoroughly as possible in a “Withdrawal/ discontinuation” form.

Should a participant wish to withdraw from receiving the allocated trial treatment, efforts will be made to continue to obtain follow-up data, with the permission of the patient or family as appropriate.

8 SAFETY

8.1 Definitions

Term	Definition
**Adverse Event (AE)**	Any untoward medical occurrence in a participant to whom a medicinal product has been administered, including occurrences which are not necessarily caused by or related to that product.
**Serious Adverse Event (SAE)**	A serious adverse event is any untoward medical occurrence that:<br>• results in death<br>• is life-threatening<br>• requires inpatient hospitalisation or prolongation of existing hospitalisation<br>• results in persistent or significant disability/incapacity<br>• consists of a congenital anomaly or birth defect<br>Other ‘important medical events’ may also be considered serious if they jeopardise the participant or require an intervention to prevent one of the above consequences.<br>NOTE: The term "life-threatening" in the definition of "serious" refers to an event in which the participant was at risk of death at the time of the event; it does not refer to an event which hypothetically might have caused death if it were more severe.

8.2 Safety monitoring

Trial participants will consent to the use of data captured by the UK Renal Registry (UKRR), NHS Digital (NHSD), Information Services Division Scotland (ISD), Patient Episode Database for Wales (PEDW) and Health and Social Care Services Northern Ireland (HSNI) data. The CTU will collect all events associated with hospital admissions from NHSD, ISD, PEDW or HSNI data as appropriate, and deaths from civil registration (NHSD) on a continuous basis. All hospitalisation-requiring and hospitalisation-associated events, and all deaths, will therefore be captured continuously directly by the CTU.

Given (a) the intensive monitoring of dialysis patients in routine clinical care, (b) the comprehensive data on clinical events recorded directly by the CTU, and c) the routine use of both high-volume HDF

and high-flux HD as part of routine NHS care, the H4RT trial will utilise the following risk-adapted safety reporting approach:

1. Adverse events will be regularly screened for:

(a) monthly during recruitment and quarterly during follow-up by the local research team and

(b) as directed by the DMC using data collected specifically for the trial and data derived from linkage to routine healthcare databases. .

2. Adverse events (AEs) and serious adverse events (SAEs), i.e. adverse events not considered to be directly related to high-volume HDF, do not require to be recorded and reported (using the standard reporting form) to the sponsor by the PI.

3. Adverse reactions (ARs), i.e. adverse events considered to be directly related to high-volume HDF but not serious, also do not require to be recorded and reported (using the standard reporting form) to the sponsor by the PI.

4. Serious adverse reactions (SARs) and suspected unexpected serious adverse reactions (SUSARs), i.e. adverse events considered to be serious and directly related (or possibly/ probably directly related) to high-volume HDF, however, do require reporting (using the standard reporting form) to the CI and the sponsor as described under 8.3 and 8.4 below. The local research team should maintain a log of SAEs that they identify and agree are not SARs. This will not need to be routinely submitted to the CI but will act as a local record of decision making and may be asked for if a site is monitored.

5. Line listings of SAEs and reported SARs & SUSARs will be reviewed monthly by the CI and submitted annually to the DMC and REC.

8.3 Recording and reporting of unexpected SARs and SUSARs

All unexpected SARs and all SUSARs occurring from the time of consent until 30 days after the end of the trial must be reported by the PI to the CI and Sponsor within 24 hours of awareness using the SAR/ SUSAR form (see Key Trial Contacts section for emails contact details t). A further review of expectedness will then be undertaken by the CI. The CI will report any reportable SAR and all SUSARs that are related to the research procedures to the Research Ethics Committee and the Sponsor within 24 hours becoming aware of the event. For each reportable SAR and SUSAR the following information will be collected:

- Full details in medical terms and case description;
- Event duration (start and end dates, if applicable);
- Action taken;

- Outcome;
- Seriousness criteria;
- Causality (i.e. relatedness to trial/intervention), in the opinion of the investigator;
- Whether the event would be considered expected or unexpected.

Each reportable SAR and SUSAR must be reported separately and not combined on one SAR/ SUSAR form. Any change of condition or other follow-up information relating to a previously reported SAR/ SUSAR should documented on the appropriate form (see Key Trial Contacts for email contact details) emailed securely to the CTU and Sponsor as soon as it is available or within at least 15 days of the information becoming available to the research team. Events will be followed up until the event has resolved or a final outcome has been reached.

8.4 Responsibilities

Adverse events will be documented and reported in accordance with North Bristol NHS Trust's Safety Reporting SOP.

8.4.1 Principal Investigator/research staff

Principal investigators (PIs) and research staff at each site will be checking for AEs and ARs when participants attend for treatment /follow-up; they will be responsible for:

- Using medical judgement in assigning seriousness, causality and expectedness. These decisions must involve a medically trained member of the research team and should be recorded on the local log (see 8.2, above).
- Ensuring that all unexpected SAEs, unexpected SARs and SUSARs are documented and reported to the Sponsor within 24 hours of becoming aware of the event and provide further follow-up information as soon as available. Ensuring that unexpected SAEs, unexpected SARs and SUSARs are chased with the Sponsor if a record of receipt is not received within 2 working days of initial reporting.
- Ensuring that all follow up data for reported SAEs, SARs and SUSARs are provided to the CI and sponsor as soon as the event ends or when any new information becomes available.

8.4.2 Chief Investigator

The chief investigator will be responsible for:

- Review the reported SAEs, SARs and SUSARs monthly, considering the overall safety of participants in the trial and updating the Sponsor, oversight committees and sites as appropriate.
- Using medical judgement in assigning seriousness, causality and expectedness of SAEs, SARs and SUSARs where it has not been possible to obtain local medical assessment.
- Using medical judgement in assigning expectedness.
- Immediate review of all reportable SAEs, SARs and SUSARs.
- Ensuring safety reports are prepared in collaboration with appropriate members of the TMG group for the main REC and DMC.
- Reporting safety information to the independent oversight committees identified for the trial (DMC and TSC).
- Expedited reporting of unexpected SAEs, unexpected SARs and SUSARs to the REC within required timelines.
- Notifying PIs of unexpected SAEs, unexpected SARs and SUSARs that occur within the trial.
- Central data collection of SAEs, SARs and SUSARs.

8.4.3 Sponsor

The sponsor will be responsible for overall oversight of the trial.

8.4.4 Trial Steering Committee (TSC)

In accordance with the Trial Terms of Reference for the TSC, this group will be responsible for periodically reviewing safety data and liaising with the DMC regarding safety issues.

8.4.5 Data Monitoring Committee (DMC)

In accordance with the Trial Terms of Reference for the DMC, this group will be responsible for periodically reviewing overall safety data to determine patterns and trends of events, or identifying safety issues, which would not be apparent on an individual case basis.

9 STATISTICS AND DATA ANALYSIS

9.1 Sample size calculation

We anticipate that at 3 years of follow-up 65% of patients on HD will have experienced our composite endpoint and we plan to detect a HR of 0.75. This effect size was agreed to be clinically significant at an investigator meeting involving patients and health care professionals. We assume any effect will be attenuated by (i) cross-over between arms (15% HD to HDF & 5% HDF to HD) and (ii) participants being allowed to take part in other trials simultaneously. To optimise recruitment and avoid excluding eligible patients because they are already participating /want to participate in other trials, an additional adjustment has been made that assumes up to half of patients in both groups will take part in another trial that assigns half of these to an intervention that reduces our composite end-point (HR=0.9), the anticipated proportion experiencing an event on HD will be 62.5% (37.5% surviving event-free) & on HDF it will be 54.1% (45.9% surviving event-free) giving a revised HR of 0.79. The number of events required to detect this difference with 90% power and a 5% significance level is 801, which requires 1348 participants in total. The primary analysis will be intention to treat and to avoid informative censoring participants will not be censored for transplant (10). Allowing for 10% loss to follow-up for other reasons we require 1527 participants and will recruit 1550.

9.2 Planned recruitment rate

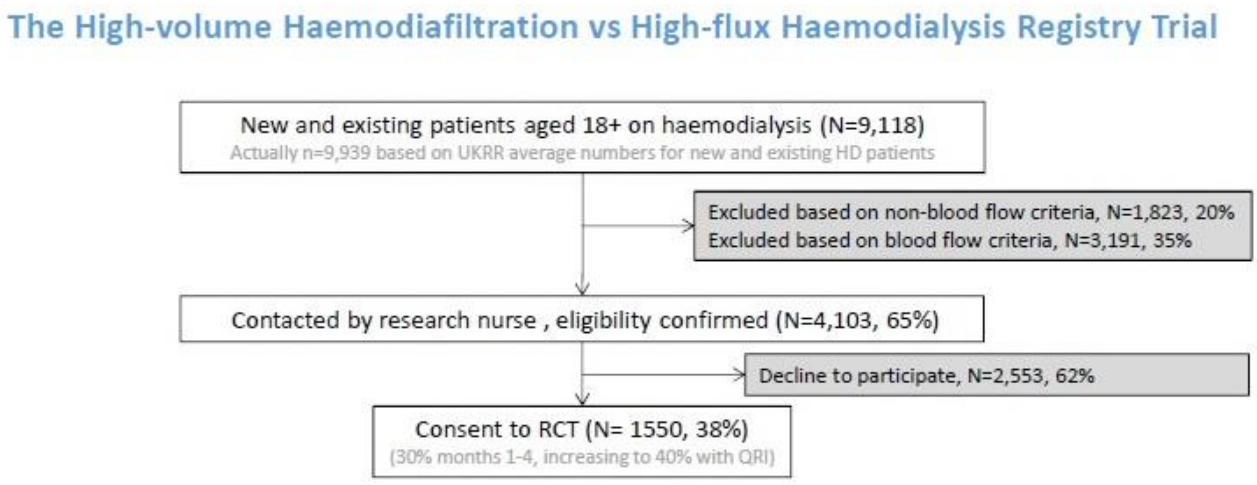

Figure 3 Screening and eligibility numbers

Recruitment will begin in all sites in month 7 and continue for at least 18 months, as required to recruit the required sample size. The number of potentially eligible patients has been estimated on the following basis:

- **Potential participants in the 20 or more participating sites**. On 31st December 2014, there were 24,166 patients on in-centre haemodialysis in the UK (44). In that year, 7,411 patients started in-centre HD (44), with a similar number starting each year. This means that each renal

unit (n=71) will have an average of 497 patients available for screening during the 18-month recruitment period, i.e. ((24,166 +(7,411 x1.5)) /71). With 20 or more sites recruiting, we would therefore expect 9,939 potential participants across all sites over at least 18 months, as required to recruit the required sample size (i.e. 497 x20), slightly more than the 9,118 required (**Figure 3**).

- **Eligibility criteria**
    - *Potential to achieve high-volume HDF*. Good vascular access in the form of an arteriovenous (AV) fistula or graft is essential for achieving high convection volumes; vascular access in the form of a dialysis catheter makes it very difficult to achieve these volumes. On 31st December 2013, 76% of HD patients in the UK were dialysing on either an AV fistula or graft (45). Considering such patients, it has been shown that 87% of those with an AV fistula can achieve substitution volumes of 21+L (i.e. convection volumes of 23+L) and 83% of those with an AV graft (14). These patients are likely to be easily identifiable in advance as the blood flow in their access – something integral to the routine dialysis prescription – will be known to be lower than required. We can therefore assume that 65% of patients on HD in a dialysis unit will be considered able to meet the target convection volume, i.e. 86% of 76%.
    - *Other exclusion criteria***.** It is estimated that a further 20% will be ineligible for other clinical reasons such as clinician predicted prognosis of less than 3 months, a living kidney donor transplant scheduled within 3 months, or transition to home haemodialysis or peritoneal dialysis planned.
      Combining these two broad categories of criteria excludes 55% (35% +20%) of potentially eligible patients, leaving 4,104 patients (i.e. 9,118 x0.45).

- **Patient agreement to participate**. Recognising the potential barriers to recruitment in this trial the QRI has been incorporated (see Section 7.7). We have anticipated an initial participation rate of 30% in months 1-4, increasing to 40% in months 5 - end of recruitment with the incorporation of lessons learned from the QRI. We anticipate a recruitment period of at least 18 months (or as long as is required to achieve the required sample size).

A screening log compiled by the BTC and QRI researcher, will document the patients assessed for eligibility for the trial, including those approached, those given the study information, and those visited by the nurse. These eligibility details, along with rates of recruitment (percentage of eligible patients agreeing to randomisation) and reasons for patients not consenting to participate will be described in reports sent monthly to the TMG. They will also be used in reviews of participating sites as required.

9.3 Statistical analysis plan

9.3.1 Summary of baseline data and flow of patients

All analysis and reporting will be in line with Consolidated Standards of Reporting Trials (CONSORT) guidelines. Primary analyses will be conducted on an intention-to-treat (ITT) basis. A full statistical analysis plan will be developed and agreed by the trial steering committee (TSC) prior to undertaking the analyses of the main trial.

Descriptive statistics will be used to determine whether there are imbalances at baseline between treatment groups and will inform any later sensitivity analyses where appropriate additional adjustment will be performed. Patient-reported outcome scores based on standardised questionnaires will be calculated based on the developers' scoring manuals and missing and erroneous items will be handled according to these manuals. Continuous measures will be presented as means and standard deviations or medians and ranges depending on their distribution. Categorical data will be presented as frequencies and proportions.

9.3.2 Primary outcome analysis

The primary endpoint in this study is a composite outcome of non-cancer death or hospital admission for infection or cardiovascular event by a minimum of 32 to maximum of 91 months follow-up. We will compare the distribution of time-to-events between the two groups using Kaplan-Meier curves and log-rank test. We will use Cox's proportional hazard model - or an alternative flexible parametric model to compare the two groups if the assumption of proportional hazards is not met - to compare the time-to-events between the two groups with adjustment for stratification variables.

A per protocol analysis will be performed with patients censored at the time of discontinuation of allocated treatment (including receipt of a kidney transplant, transition to home dialysis, receipt of the alternative form of HD/HDF on the last session of two sequential months, and patient choice to come off allocated treatment), with adjustment made for baseline characteristics. Recognising biases inherent to per protocol analyses when compliance is not random, we will also consider appropriate alternative causal inference approaches to estimate the treatment effect in compliers.

9.3.3 Secondary outcome analysis

The secondary outcomes of all-cause mortality, non-cancer mortality, cardiovascular mortality, infection mortality will be analysed and reported in a similar manner to the primary outcome as described in section 9.3.2.

Cardiovascular and infection-related hospitalisations and MRSA and MSSA infections are all recurrent events and analyses of such outcomes should account for informative censoring due to death. We will therefore use joint frailty models (JFMs) as these simultaneously analyse recurrent events (infections or hospitalisations) and time to death while estimating distinct hazard ratios. Appropriate repeated measures regression models for patient-reported outcomes will be chosen based on the distribution of the data and will adjust for stratification variables and values of the outcome at the time of randomisation. A similar repeated measures regression approach will be taken to the following other repeated measure outcomes: time to recover after each dialysis session, haemoglobin levels, erythropoiesis stimulating agent dose, calcium levels, phosphate levels, PTH levels, albumin levels, and phosphate binder dose.

9.4 Subgroup analyses

We will conduct pre-planned subgroup analyses to investigate any differential effects according to baseline urine volume (a surrogate for residual kidney function), diabetic status, line, weight at baseline, age and the experience of sites in achieving high-volume HDF. Subgroup analyses will be conducted by including an interaction term between allocated treatment and baseline characteristic in regression models then using the likelihood ratio test comparing this model with one excluding the interaction term. Urine volume and age will be included in the model as continuous variables.

9.5 Adjusted analysis

All analyses will be adjusted for stratification variables and, in the case of patient-reported outcomes, we will also adjust for the value of the outcome pre-randomisation. For all statistical models used the underlying assumptions will be checked using standard methods. If assumptions are not valid then alternative methods of analysis will be sought.

9.6 Interim analysis

A formal interim analysis of all-cause mortality will be conducted using available data and discussed with the DMC. A one-sided p-value<0.025 for treatment differences (mortality (HD)<mortality (HDF)) would be considered a meaningful difference to be explored further. The analysis will take place half-way through the study although the exact timing of the analysis will be set by the DMC who will be guided by recruitment patterns and progress in obtaining permissions for relevant linkages.

	1. The number of participants recruited is at least 85% of what would be expected if all 20 centres recruiting from the first day of the recruitment period at the expected rate (i.e. at least 388 of the required 456). This is regardless of the number of sites recruiting. OR<br>2. The number of sites recruiting is at least 85% of what would be expected (i.e. at least 17 of the required 20) AND the rate of participant recruitment per active site month is at least 85% of what would be expected (i.e. at least 3.4 of the required 3.8).
	1. The number of participants recruited is 60-84% of what would be expected were all 20 centres recruiting from the first day of the recruitment period at the expected rate (i.e. 274-387 of the required 456). This is regardless of the number of sites recruiting. OR<br>2. The number of sites recruiting is 60-84% of what would be expected (i.e. 12-16 of the required 20) AND the rate of participant recruitment per active site month is 60-84% of what would be expected (i.e. 2.3-3.3 of the required 3.8).
	1. The number of participants recruited is less than 60% of what would be expected were all 20 centres recruiting from the first day of the recruitment period at the expected rate (i.e. less than 273 of the required 456). This is regardless of the number of sites recruiting. AND<br>2. The number of sites recruiting is less than 60% of what would be expected (i.e. less than 11 of the required 20) AND the rate of participant recruitment per active site month is less than 60% of what would be expected (i.e. less than 2.2 of the required 3.8).
NOTE: The participant recruitment rate of 3.8 participants per active site month for the first six months of recruitment is slightly lower than the rate required across the whole trial, 4.3. This reflects the anticipated lower recruitment in the early months that the QRI work is intended to improve – 30% in months 1-4, increasing to 40% in months 5 - end of recruitment.	

In all cases, we will also report the percentage of HD patients that meet the eligibility criteria and then the percentage who consent to randomisation. To assess the generalisability of participants the characteristics of consenting participants and non-consenting and routine HD patients (from screening logs and UKRR data, respectively) will be compared. We will also report preliminary data on event rates observed in the trial population – death rates, cardiovascular and infection hospital admission rates, dropout rates, transfer to a centre not offering HDF, transfer to a different treatment modality such as peritoneal dialysis or transplantation – and how close these are to the assumptions made in the sample size calculation.

In the case of an amber result, a more detailed breakdown of site and participant recruitment would be provided, along with a review of event rates and crossover rates and a report from the QRI re the barriers to recruitment.

At the first DMC meeting, the committee will agree on its charter of operations and advise on the criteria for the need for interim analyses and adoption of formal stopping rules for efficacy or safety. The DMC will be responsible for assessing safety and efficacy; they will be responsible for

recommending stopping the trial at any time if there are significant safety or ethical issues. Judgements will be made at their discretion.

Any interim statistical analyses by study arm will be performed by the study statistician blinded to treatment allocation. They will report blinded data to the DMC who will have unblinded access to all data if they have concerns about the safety of the RCT and will discuss the results of the interim analyses with the TSC in a joint meeting. The TSC will then report to the central ethics committee.

A detailed statistical analysis plan will be developed for the approval of the TSC and will be finalised before any interim analyses are undertaken for the DMC.

9.7 Subject population

The subject population includes all adult patients on in-centre, maintenance HD or HDF for ESKD. Our exclusion criteria are:

- Lacks capacity to consent;
- Clinician predicted prognosis of less than 3 months;
- Started maintenance HD or HDF within 4 weeks;
- Transition to living kidney donor transplant or home dialysis scheduled within 3 months;
- Not suitable for high-volume HDF for other clinical reasons such as dialysis less than thrice weekly or unlikely to achieve sufficient blood flow rates with current vascular access, or prior intolerance of HDF.

All randomised participants will be included in intention-to-treat analyses.

A per protocol analysis will also be conducted of the primary outcome where patients are censored at the time of cross-over and adjustment will be made for baseline characteristics.

9.8 Procedure(s) to account for missing or spurious data

Where missing data exist the frequency of missing data will be indicated and if the amount of missing data differs substantially between treatment groups (>10%) potential reasons will be explored. Sensitivity analyses will be conducted (including the use of multiple imputation methods where assumptions are met) to examine the influence of missing data on the key trial findings.

9.9 Other statistical considerations

A detailed statistical analysis plan will be developed for the approval of the TSC prior to analysis. Any deviation(s) from the approved plan will be described and justified to the TSC for their approval.

9.10 Economic evaluation

There is limited evidence on the cost-effectiveness of high-volume HDF compared to high-flux HD. Previous work alongside the CONTRAST trial which compared online HDF with low-flux HD has not

provided conclusive evidence (24). Cost-utility data were only collected on 409 of 714 patients randomised in CONTRAST. The CONTRAST trial suggested that HDF was marginally more costly than low-flux HD over a 3-month period (€88,622 vs €86,086), primarily due to the higher cost of disposable equipment and water purity control. Over 5 years of follow up quality adjusted life years (QALYs) were also marginally higher (2.40 vs 2.34) in patient receiving HDF. The incremental cost per quality-adjusted life year (QALY) of HDF versus HD was €287,679, indicating that it would not be cost-effective. A subsequent post-hoc subgroup analysis (n=130) from a Canadian CONTRAST site that achieved high-volume HDF in the majority of its participants reported significantly higher QoL in patients on high-volume HDF. They concluded that the additional costs of high volume HDF were largely due to increased survival and that these costs were justified by better outcomes ($CAN 32,112 per QALY gained) (25). Due to the high costs of RRT, it is very important to provide more definitive evidence on the cost-effectiveness of high volume HDF to determine whether it should be more widely adopted on the NHS.

The primary economic analysis will take an NHS perspective in order to minimise the participant burden and increase efficiency of the RCT. Additional analyses will explore any impact of treatment on residential care. . This will be based on brief resource use questions collected directly from patients. The analysis will include a 'within trial' analysis estimating cost-effectiveness during the trial follow up period. The costs associated with high-flux HD and high-volume HDF will be determined by calculating the incremental cost of equipment, staff-time, and materials/consumables used in performing dialysis. Healthcare resource use, including hospital visits and admissions, GP and community care contacts, and medication use will be obtained from linked hospital statistics in England, Scotland, and Wales, as necessary, the UK Renal Registry, and patient self-completed questionnaires and evaluated using published unit cost sources .Should differences in any component of the primary outcome between the study groups be evident at the end of trial follow-up , resulting in uncertainty over the longer-term cost-effectiveness estimates of HDF, we will develop a probabilistic decision analysis model to extrapolate cost-effectiveness estimates over patient lifetimes. Further details of the economic analysis will be provided in a publicly available health economic analysis plan.

Source data for the economic evaluation include UKRR, hospital statistics (HES, PEDW, ISD), and patient reported quality of life and healthcare and medication use as described in previous sections of the protocol.

We will annuitize the capital costs (e.g. machine) of HDF and HD based on purchase price, useful life, discount rates, resale value and estimate the cost per dialysis visit based on dialysis time and annual throughput (46). We anticipate that patients receiving HDF will have more

advanced dialysis machines capable of monitoring blood flow and concentration, although this will not be the case at all sites. We will obtain typical unit costs for consumables (e.g. blood lines, ultra-pure water, reinfusion line, microbiological testing, etc.) from a survey of participating dialysis units. We will use observations at purposively selected sites to collect data on resources (e.g. machine set up time) not routinely recorded. Using these data we will micro-cost dialysis sessions using methods similar to those previously published by our research team (47). Medication costs (e.g. Erythropoiesis stimulating agents and phosphate binders) will be estimated from the British National Formulary. National unit costs will be used to value hospitalisations, GP and community care (48, 49).

As HDF and HD require dialysis with similar frequency and duration, we do not expect any major impact on patient/family expenses. However, there is a small possibility that patients may need more residential care in one arm over the other. Therefore, our analysis focusses on the NHS perspective and we will include a secondary analysis looking at NHS and residential care. QALYs will be estimated from EQ-5D-5L responses and mortality data during follow up, accounting for any baseline differences in EQ-5D-5L scores. Missing cost and QALY data may be imputed using simple or multiple imputation methods. Cost and QALY data will be combined to calculate an incremental cost-effectiveness ratio (ICER) and incremental net monetary benefit (INMB) statistic (50). For each individual i, the NMB is the willingness to pay for a QALY, λ, multiplied by the patient outcome Ei (i.e. QALYs), minus the cost of health care Ci; NMBi = λ Ei–Ci. In the primary analysis we will estimate whether HDF is cost-effective at the established NICE threshold of £20,000 per QALY gained. Uncertainty in the point estimate of cost per QALY will be quantified to calculate confidence intervals around the ICER and INMB. The probability that HDF is cost-effective at various 'willingness to pay for a QALY' thresholds and in the pre-specified subgroups (residual renal function and age) will be depicted using a cost-effectiveness acceptability curve (51).

If appropriate, a probabilistic decision analysis model will be developed and populated with many parameters estimated directly in the RCT (e.g. short-term hazard rates and ratios for mortality, peritoneal dialysis, transplant, hospitalisation; costs and utility scores for HDF and HD). Other parameters (e.g. long-term hazard rate of mortality, peritoneal dialysis, transplant, hospitalisation after HD) will be estimated based on registry data and a rapid review of the epidemiological literature of longitudinal studies and implemented using a Markov model. The model will predict the time-variant probability of patient transition through a small number of health states (e.g. HD/ HDF, PD, transplant, death) and the costs and quality of life associated with those health states. Access to HES-CR linked individual patient registry data will be a

significant advantage in this regard, allowing us to fit the most appropriate models for long-term survival and disease progression.

10 DATA HANDLING

10.1 Data collection tools and source document identification

Baseline data will be entered directly into a case report forms (CRF) and sent securely (by post or electronically) no more than 4 weeks after the baseline visit to the CTU for entry into the database. Baseline patient questionnaires (PQs) will be administered by the research staff at the baseline visit and returned to the CTU for entry into the database; thereafter, PQs will be sent out 6-monthly by the CTU to the patient with an enclosed cover letter signed by the site principal investigator and research nurse and returned directly to the CTU. If a patient questionnaire is not returned, up to 2 reminders will be sent. Where appropriate questionnaires can be administered by telephone from the CTU to get responses from the patient to the EQ5D questions.

Standardised tools are being used:

- Co-morbidity: Davies co-morbidity Score (52) & Charlson co-morbidity index (53)
- Quality of life: EQ-5D-5L (54), and DSI (55).

A central administrative database will be set up by BTC that prompts the CTU when PQ forms are due.

PIs must keep records of all participating patients (sufficient to link records e.g., CRFs and hospital records), all original signed informed consent forms and copies of the CRF pages.

10.2 Data handling and record keeping

10.2.1 Database platforms

All administrative and clinical study data will be stored in REDCap. REDCap is a secure, web-based electronic data capture (EDC) system designed for the collection of research data. The system has been developed and supported by Vanderbilt University. Bristol Trial Centre (BTC) at the University of Bristol (UoB) has set up its own infrastructure so that all systems are hosted at UoB.

A Relation Database Management System will be used to provide integration services between administrative and clinical databases. These data will be stored here, to support the workflow of the study team. These data will be not made available for analysis.

10.2.2 Administrative Data

The Administrative data will be kept in a secure database that is only accessible from within the UoB firewall. All users will require (at least honorary) contracts with UoB in order to access it.

10.2.3 Clinical Data

The clinical data will be stored on a separate server to the administrative data. Anonymized clinical data is linked by a participant ID. Email addresses are collected as they are essential for the correct functioning of the survey feature. The 'Email Address' field is flagged as an identifier and not included in the export for the statistician, so the data set can be considered pseudonymised at export and doesn't need further processing.

10.2.4 System Design

A combination of field type validation, data ranges, logic and thorough testing is used to ensure the quality of the data collected.

10.2.5 Data Entry

Admin Data is entered directly via the website. Clinical data can either be entered this way or by participants completing online surveys.

10.2.6 Reporting and Export

Reporting and export procedures for data downloads to common statistical packages (SPSS, SAS, Stata, R) are provided

10.2.7 Storage

Data are stored in secured UoB servers subject to standard UoB security procedures. The full databases are backed up daily. Additionally, changes are logged every 5 minutes. Disaster/recovery plans are in place as part of the Service Level Agreement (SLA) we have with IT Services.

10.2.8 Security

In order to access the application directly, study team users will be added to the system (following request from the Trial Manager) by the BTC Data Manager. Data access can be restricted by User roles. This facility can be used to avoid unblinding the statistician if necessary. It is the Trial Manager's responsibility to add the user to a specific project and role.

10.2.9 Auditing

A full audit log catalogues individual changes with date/time, old value, new value and the identity of the user who made the change.

10.3 Access to Data

10.3.1 Source data

The PI will allow monitors from the sponsor (NBT R&I), persons responsible for the audit, representatives of the Research Ethics Committee and of the Regulatory Authorities to have direct access to source data/documents.

10.3.2 Anonymised trial data

The Senior IT Manager (in collaboration with the Chief Investigator) will manage access rights to the data set. Prospective new users must demonstrate compliance with legal, data protection and ethical guidelines before any data are released. We anticipate that anonymised trial data will be shared with other researchers to enable international prospective meta-analyses.

10.4 Archiving

This trial will be sponsored by North Bristol NHS Trust, with University of Bristol as the data custodian. Hard copies of completed case report forms will be kept for 15 years following the end of a study to enable audit of data used in publications. These will be kept at the University of Bristol for this time and then destroyed.

11 MONITORING, AUDIT AND INSPECTION

The study will be monitored in accordance with North Bristol NHS Trust's Monitoring SOP. All trial related documents will be made available on request for monitoring and audit by North Bristol NHS Trust, the Research Ethics Committee and available for inspection by other licensed bodies. The monitoring plan will be developed and agreed by the sponsor.

Monitoring and audits undertaken by North Bristol NHS Trust, under their remit as sponsor, or individuals appointed responsibility for monitoring on behalf of the Trust, will ensure adherence to GCP and the NHS Research Governance Framework for Health and Social Care (2nd edition). Remote monitoring will be conducted based on information submitted by sites and analysis of the trial database. Site visits will then be initiated using a risk-based approach.

12 ETHICAL AND REGULATORY CONSIDERATIONS

12.1 Research Ethics Committee (REC) review and reports

Ethical and Health Research Authority (HRA) approval will be sought through the HRA for the trial and the qualitative work embedded within the trial. We believe the proposed research does not pose any specific risks to individual participants nor does it raise any untoward ethical issues. As with all trials, the main benefit of participating is an altruistic one to improve care for subsequent patients with kidney failure. As a registry trial, surveillance will be according to routine care. There are no known additional risks for patients in participating, with HDF already a routine part of care in ~15% of cases in the UK and being scaled up. A letter of invitation to participate in the study and a patient information sheet will be developed in collaboration with the PAG and in line with guidance from the HRA. The patient information sheet will provide clear details of the anticipated risks and benefits of taking part in the trial and the study interventions, and may be modified by the findings from the QRI work. The risk and benefits of the study will also be discussed with the local research staff and nephrologists as part of the process of providing written informed consent.

All staff doing specific research activities will be required to complete training in Good Clinical Practice. Informed consent to participate in the trial will be sought and obtained according to Good Clinical Practice guidelines. Informed signed consent forms will be obtained from all participants in all centres, by an appropriately trained individual. Participants will be given sufficient time to accept or decline involvement and will be free to leave the study at any time. Participants who cannot give informed consent (e.g. due to their mental state) will be not be eligible. The participants will be asked to consent to: participation; randomisation; follow up; contact in the future about this and other research; electronic tracing using NHS data; and data linkage with routine NHS data sources.

All research will be performed in accordance with the recommendations guiding biomedical research involving human subjects adopted in the 18th World Medical Assembly, Helsinki, Finland.

Health Research Authority approval will be sought, where appropriate, for any analyses relating to UK Renal Registry data collected under section 251 of the NHS Act 2006 on nonparticipating patients.

All correspondence with the REC will be retained in the Trial Master File/Investigator Site File. An annual progress report will be submitted to the REC within 30 days of the anniversary date on which the favourable opinion was given, and annually until the trial is declared ended. The CI will notify the REC of the end of the study and if the study is ended prematurely (including the reasons for the premature termination). Within one year after the end of the study, the CI will submit a final report with the results, including any publications/abstracts, to the REC.

12.2 Peer review

The proposal for this trial has been peer-reviewed through the NIHR HTA peer-review process, which includes independent expert and lay reviewers.

In addition, the protocol has been reviewed by the Trial Management Group and the Sponsor.

12.3 Public and Patient Involvement

Potential topics for a registry-based efficient study were discussed at the Registry's Patient Council and the topic of "effectiveness of haemodiafiltration" prioritised. The Patient Council and local Kidney Patient Association (both ~15-20 dialysis and former dialysis/ transplant patients) told us they wanted to know whether HDF improves survival, symptoms and quality of life, and whether it is safe. Some raised concerns about its environmental impact. PPI co-applicant Mrs Abbott has attended planning meetings and helped draft this application.

Patients and the public will take an active role in the running of the trial through:

- *The Trial Steering Committee:* PPI chair, Mr Kristian Law will sit on the TSC.
- *The Patient Advisory Group:* This will be chaired by Mr Kristian Law and provide advice, support and oversight of patients' involvement throughout the study. This group will meet twice in the first and last year of the trial and annually in other years.

Mr Kristian Law, with help from other members of the PAG, will develop patient information and advise on study design to optimise its acceptability to patients. The PAG will also act as a point of contact for patients. Progress and results from the study will be presented to the group and patient interpretation sought. They will also advise on the best way to disseminate the study findings to patients, including the production of plain English summaries.

Members of the PPI group will be involved in a number of ways – face-to-face meetings, workshops for more in-depth work and email for reviewing documents – in the following activities:

- Designing information and consent sheets and in designing the recruitment process to maximise their accessibility from a patient perspective
- Acting as a point of contact for participants and potential-participants throughout the study
- Reading summaries of the QRI findings to ensure that patient concerns are adequately reflected in the analysis
- Developing plain English summaries of the findings that can be used by patients and cares to assist them in making evidence based treatment decisions and developing a dissemination policy.

12.4 Regulatory Compliance

Before any site can enrol patients into the trial, the CI/PI or designee will obtain confirmation of capacity and capability for each site.

For all amendments the CI/PI or designee will confirm with the Sponsor, the HRA (+/- REC) and sites' R&D departments that permissions are ongoing.

12.5 Protocol compliance

There will be no prospective, planned deviations or waivers to the protocol. Accidental protocol deviations can happen at any time, but they must be adequately documented on the relevant forms (see Key Trial Contacts section for link to website) and reported to the CI and Sponsor immediately. Deviations from the protocol which are found to frequently recur are not acceptable, will require immediate action and could potentially be classified as a serious breach.

12.6 Notification of Serious Breaches to GCP and/or the protocol

A "serious breach" is a breach which is likely to effect to a significant degree:

a) the safety or physical or mental integrity of the subjects of the trial; or
b) the scientific value of the trial

The sponsor must be notified immediately of any case where the above definition applies during the trial conduct phase. They will assess the seriousness of any breach as per the appropriate SOP (see Key Trial Contacts section for link to website).

12.7 Data protection and patient confidentiality

The University of Bristol will be the data custodian. All data held in Bristol will conform to the University of Bristol Data Security Policy and in Compliance with the Data Protection Act 1998.

Data collected on paper case report forms at study centres or as questionnaires from participants will be identifiable only by participant study number. This will be transported by post or securely electronically to the H4RT study office at University of Bristol, and stored in a secure locked cabinet in a locked room.

Data obtained by paper will also be entered onto and maintained on an SQL Server database system maintained by University of Bristol Information Services. Information capable of identifying individuals and the nature of treatment received will be held in the database with passwords restricted to H4RT study staff. Information capable of identifying participants will not be removed from University of Bristol or clinical centres or made available in any form to those outside the study.

Patient identification codes will be held by the University of Bristol for 15 years, all other data sources will be stored for 15 years after the close of the study. Personal data (e.g. name and address, or any

data from which a participant might be identified) will be withdrawn from the study if this is requested by a participant.

Interviews and recruitment appointments will be recorded on an encrypted digital recorder which will be locked in a secured cabinet at the School of Social and Community Medicine. Recordings will be transferred onto a computer as soon as possible after each interview, and stored only in a password protected drive maintained by the University of Bristol. Only the qualitative researchers working on this study will have access to this drive.

Recordings and transcriptions will be named with a study-assigned participant number, centre initials, and the date of recording. There will be no participant identifiers in files, databases, or transcripts, which will only be labelled with study assigned participant numbers. Coding keys matching the name of the participants with their study participation number will be stored in a password protected spreadsheet, which will be maintained and only accessed by the qualitative researchers. All recordings will be coded and securely transferred to a University of Bristol approved transcription company or transcriber that has signed the required confidentiality agreements. All transcripts will be anonymised upon receipt.

All electronic data files will be saved in a secured computer and to a password protected University of Bristol network space, in accordance with the University of Bristol’s data security policies.

All nonessential data will be wiped upon completion of the study. Essential documents will be kept for up to 15 years, after which they will be deleted and all copies destroyed in accordance with the University of Bristol’s secure erasure of data policy.

The anonymised interview data (transcripts only) will be uploaded to a ‘controlled access’ data repository, subject to individual written informed consent from the participants. This has been fully explained in the information sheet, and is requires participants to initial a specific statement on the consent form (if they agree).

12.8 Financial and other competing interests for the chief investigator, PIs at each site and committee members for the overall trial management

The research team and all PIs must disclose any ownership interests that may be related to products, services, or interventions considered for use in the trial or that may be significantly affected by the trial. Competing interests will be reported in all publications and in the final report.

12.9 Indemnity

The necessary trial insurance is provided by the Sponsor. North Bristol NHS Trust holds standard NHS Hospital Indemnity and insurance cover with NHS Litigation Authority for NHS Trusts in England,

which apply to this trial. The Patient Information Sheet provides a statement regarding indemnity for negligent and non-negligent harm.

12.10 Amendments

The Sponsor will determine whether an amendment is substantial or non-substantial. All amendments will be processed through the HRA and where appropriate the REC. If applicable, other specialist review bodies (e.g. CAG) will be notified about substantial amendments in case the amendment affects their opinion of the study. Amendments will also be notified to NHS R&D departments of participating sites to confirm ongoing capacity and capability to deliver the study.

12.11 Post trial care

Following the end of the trial, continued provision of high-flux HDF will be at the discretion of the normal care team and is likely to depend on the trial results. Participants will be informed of this in the written information given to them when they are considering entering the trial.

12.12 Access to the final trial dataset

Anonymous research data will be stored securely and kept for future analysis. Members of the TMG will develop a data sharing policy consistent with University of Bristol policy and reviewed by the TSC. Data will be kept anonymous on secure access computers, and access will be via written confidentiality and data sharing agreements (DSA) with the CI (or his appointed nominee), supervised by the CI with the involvement of other members of the research team. All requests for data release outside of the planned analyses will be considered by the TSC. Any request approved will be covered by a written Data Sharing Agreement, detailing limitations of use, transfer to 3rd parties, data storage and acknowledgements. The person applying for use of the data will be scrutinized for appropriate eligibility by members of the research team. All requests will require their own separate REC approval prior to data being released. Data will not be released prior to analyses for purposes that might detrimentally affect the trial integrity

13 DISSEMINATION POLICY

A comprehensive plan for disseminating H4RT results will be developed by TMG which will include PPI co-applicants. The results of the study will be published in academic journals and all participants will be offered a plain English summary of the main findings of the study. Meetings will be arranged with stakeholders to consider the implications of the results and how they will most effectively be translated into clinical practice.

On completion of the trial a final report will be prepared for the Funder (NHR HTA) and once approved made publicly available on their website. The Funder needs formal notice in advance of all publications and the Funder and Sponsor need to be acknowledged within the publications.

With HDF use increasing in Europe and almost non-existent in the USA, the results of the trial have the potential to be truly practice changing, with the main beneficiaries being:

- Patients and their families – health outcomes
- Health professionals – practice/ behaviour change and service development
- Hospital managers and commissioners – capacity building, investment / disinvestment of scarce
- Resources and policy decision making
- Industry partners – informing dialysis technology development
- Society – providing information on an environmental impact.

Study progress and results will be disseminated through the existing communication channels of the UK Renal Registry and the UK Renal Association. Both have active twitter accounts with 1.4k and 1.7k followers, respectively. An H4RT twitter account will be set up to keep interested patients, carers, clinicians, managers and policy makers up-to-date with trial progress. The Registry also writes pieces each month for the Renal Association's e-newsletter to all members. The lessons from this trial will be very relevant for designing future efficient trials in dialysis, transplant and indeed chronic kidney disease and these can be fed back through the UK Kidney Research Consortium Dialysis Study Group and the recently established Trials Group on which several of the co-applicants sit.

Representatives from the British Kidney Patient Association and National Kidney Federation have worked with us on this bid and they too are active on social media and have established channels for communicating the progress and findings of the study to patients such as regular newsletters, a network of kidney patient associations and annual meetings.

Once finalised the protocol will be published in an open access journal. With the key findings likely to be practice changing and of interest to a wide range of clinicians and policy makers, academic

publications will be of interest to high impact journals such as the BMJ, the New England Journal of Medicine and the Journal of the American Medical Association. Findings will be presented at leading nephrology conferences in Europe (the ERA-EDTA Annual Congress) and North America (The American Society of Nephrology Kidney Week) as well as at the UK Kidney Week, co-hosted by the Renal Association and the multi-disciplinary British Renal Society. Findings will also be used to inform future iterations of the NICE-approved UK Renal Association clinical guidelines and the European Renal Best Practice clinical guidelines.

13.1 Authorship eligibility guidelines and any intended use of professional writers

The final trial report will be written by the CI with support from the TMG and all co-investigators. All TMG members and co-investigators who have contributed to the design, conduct analysis and write up will be offered authorship on the final report.

On manuscripts arising from the trial, authorship will be on an individual authorship basis (rather than group authorship basis) with inclusion based on the recommendations of the International Committee of Medical Journal Editors will be developed.

14 APPENDICES

14.1 Appendix 1 – Assessments and follow-up

**Procedures**	**Data**	**Screening**	**Baseline** Face-to-face visit 1	**Treatment Phase** **Follow Up** (min = 32 month, max = 91 months) No visits		**Event based**
				Linkage	Patient Questionnaire (6 monthly)	
Eligibility assessment		√				
Informed consent			√			
Randomisation			√			
Demographics	Age, sex, ethnicity, marital status, education level, smoking history.		√			
Clinical (1)	Primary renal disease, date first seen by nephrologist, co-morbidities, dietary restrictions, 24-hour urine volume.		√			
Clinical (2)	RRT treatment history, prescribed medication (including erythropoiesis stimulating agents and phosphate binders),		√	√		
Physical assessment (1)	Height, heart rate.		√			
Physical assessment (2)	Weight, systolic and diastolic blood pressure		√	√		
Resource use (1)	Day case and inpatient hospital admissions (including surgical procedures performed),		√	√		
Resource use (2)	Nursing home/residential home days/hospice days, other hospital outpatient services and primary care & community services in the last 6 months.		√		√	
Laboratory tests	Creatinine, urea, Kt/V, urea reduction ratio, albumin, haemoglobin, haematocrit, mean corpuscular volume, sodium, potassium, bicarbonate, corrected calcium, phosphate, c-reactive protein, intact parathyroid hormone, total cholesterol. (From the date of the study visit or the closest date prior to the study visit.)		√	√		
Patient reported	EQ-5D-5L, and DSI and time to recovery (5).		√		√	
SAE reporting						√

14.2 Appendix 2 – Risk

Risks associated with trial interventions

☐ LOW ≡ Comparable to the risk of standard medical care

☒ MODERATE ≡ Somewhat higher than the risk of standard medical care

☐ HIGH ≡ Markedly higher than the risk of standard medical care

Justification:

As high-volume HDF involves infusing an additional 20-25L of water back into the patient x3 per week, x52 weeks per year, water quality is crucial to patient safety and has to meet "Sterile dialysate" standards (i.e. bacterial limits <10-6 CFU/mL & endotoxin limits <0.03 EU/mL). Technological developments over the past decade now make it possible to produce such water "on-line", i.e. continuously in the renal unit, and almost all renal units in the UK are providing on-line HDF as standard care to at least some patients. There remains, however, a small risk of blood stream infection being introduced as part of the HDF process. This risk is mitigated by renal units monitoring water quality on a regular basis. Any blood stream infections that are believed to be related to the HDF will be reported as SARs.

What are the key risks related to therapeutic interventions you plan to monitor in this trial?		How will these risks be minimised?		
Intervention	Body system/Hazard	Activity	Frequency	Comments
High-volume HDF	Blood stream infection	Water quality monitoring in renal units	Monthly	As per UK Renal Association Guidelines (6)

These risks will also be considered by the DMC and incorporated into data updates they request.

14.3 Appendix 3 – Study management / responsibilities

14.3.1 Role and responsibilities of trial management committees/groups and individuals

Overall project management

The Chief Investigator (CI) will take overall responsibility for managing the various components of the trial and will meet at least monthly with the leads for each component. In years 1-2 the CI will be establishing the trial, supported by the trial manager and lead renal research nurse. From a technical and strategic perspective, the CI will be advised and supported by Dr Lane and Professor Donovan.

The Clinical Trial*:* The BTC is a UK Clinical Research Collaboration (UKCRC) registered trials unit who will manage the trial on a day-to-day basis.

Patient Advisory Group

A Patient Advisory Group will meet biannually in year 1 and 5 and annually in years 2, 3 and 4. This group will be co-chaired by the PPI co-applicant.

Trial Management Group

A Trial Management Group (TMG) will meet at least once each quarter in the first 24 months, then 6 monthly to review progress, with potential for additional ad hoc meetings, as required/indicated. This will be chaired by Professor Fergus Caskey (CI) and will consist of representatives from the study office including the sponsor and relevant co-applicants, including PPI co-applicants, and the CTU. Meetings will be in person and by teleconference to maximise attendance.

Trial Steering Committee

The role of the Trial Steering Committee (TSC) is to monitor and supervise the progress of the trial on behalf of the Sponsor and Funder and to ensure that the project is conducted to the rigorous standards set out in the Department of Health's Research Governance Framework for Health and Social Care and the Guidelines for Good Clinical Practice. The TSC will comprise and independent chair, 5 additional independent members. The independent members will cover expertise in statistics, trials and haemodialysis. The trial manager and Professor Fergus Caskey (CI) will also be formal members of the TSC, maintaining its membership independence at 75% and a PPI representative will be nominated. Observers may also attend, as may other members of the TMG or members of other professional bodies, at the invitation of the Chair. The TSC will meet for the first time by month 6 of the trial and then 6 monthly.

Data Monitoring (and Ethics) Committee

Independence

For the TSC and DMC, independence is defined by the NIHR HTA as follows:

- *Not part of the same institution as any of the applicants or members of the project team;*
- *Not part of the same institution that is acting as a recruitment or investigative centre;*
- *Not related to any of the applicants or members of the project team;*
- *For the chair only, not an applicant on a rival proposal.*

An independent Data Monitoring Committee (DMC) will be appointed prior to the commencement of recruitment with the purpose of reviewing the data at pre-specified intervals to advise the TSC and the sponsor regarding patient safety and the ethical running of the trial. The DMC will monitor the trial every 6-12 months, depending on the phase of the trial, either in person or electronically. It includes an independent chair and independent members with clinical and methodological expertise. A DMC monitors accumulating trial data for quality, completeness and patient safety, and comprises of an independent chair and five other independent members, and the CI. It reports to the chair of the TSC. The DMC charter is available from the corresponding author. The CI will not attend closed meetings of the DMC, but may be invited to attend open meetings or open parts of meetings.

15 REFERENCES

1. Gilg J, Pruthi R, Fogarty D. UK Renal Registry 17th Annual Report: Chapter 1 UK Renal Replacement Therapy Incidence in 2013: National and Centre-specific Analyses. Nephron. 2015;129 Suppl 1:1-29.
2. Rao A, Casula A, Castledine C. UK Renal Registry 17th Annual Report: Chapter 2 UK Renal Replacement Therapy Prevalence in 2013: National and Centre-specific Analyses. Nephron. 2015;129 Suppl 1:31-56.
3. Steenkamp R, Rao A, Roderick P. UK Renal Registry 17th Annual Report: Chapter 5 Survival and Cause of Death in UK Adult Patients on Renal Replacement Therapy in 2013: National and Centre-specific Analyses. Nephron. 2015;129 Suppl 1:99-129.
4. Fotheringham J, Fogarty DG, El Nahas M, Campbell MJ, Farrington K. The mortality and hospitalization rates associated with the long interdialytic gap in thrice-weekly hemodialysis patients. Kidney international. 2015;88(3):569-75.
5. Fukuhara S, Lopes AA, Bragg-Gresham JL, Kurokawa K, Mapes DL, Akizawa T, et al. Health-related quality of life among dialysis patients on three continents: the Dialysis Outcomes and Practice Patterns Study. Kidney international. 2003;64(5):1903-10.
6. Mactier R, Hoenich N, Breen C. UK Renal Association Guidelines - haemodialysis 2009 [Available from: http://www.renal.org/guidelines/modules/haemodialysis#downloads.
7. Vartia A. Effect of treatment frequency on haemodialysis dose: comparison of EKR and stdKt/V. Nephrol Dial Transplant. 2009;24(9):2797-803.
8. Neirynck N, Vanholder R, Schepers E, Eloot S, Pletinck A, Glorieux G. An update on uremic toxins. International urology and nephrology. 2013;45(1):139-50.
9. Peters SA, Bots ML, Canaud B, Davenport A, Grooteman MP, Kircelli F, et al. Haemodiafiltration and mortality in end-stage kidney disease patients: a pooled individual participant data analysis from four randomized controlled trials. Nephrol Dial Transplant. 2016;31(6):978-84.
10. Mostovaya IM, Blankestijn PJ, Bots ML, Covic A, Davenport A, Grooteman MP, et al. Clinical evidence on hemodiafiltration: a systematic review and a meta-analysis. Seminars in dialysis. 2014;27(2):119-27.
11. Susantitaphong P, Siribamrungwong M, Jaber BL. Convective therapies versus low-flux hemodialysis for chronic kidney failure: a meta-analysis of randomized controlled trials. Nephrol Dial Transplant. 2013;28(11):2859-74.
12. Nistor I, Palmer SC, Craig JC, Saglimbene V, Vecchio M, Covic A, et al. Haemodiafiltration, haemofiltration and haemodialysis for end-stage kidney disease. Cochrane Database Syst Rev. 2015;5:CD006258.
13. Agar JW. Reusing and recycling dialysis reverse osmosis system reject water. Kidney international. 2015;88(4):653-7.
14. Marcelli D, Scholz C, Ponce P, Sousa T, Kopperschmidt P, Grassmann A, et al. High-volume postdilution hemodiafiltration is a feasible option in routine clinical practice. Artificial organs. 2015;39(2):142-9.
15. Susantitaphong P, Altamimi S, Ashkar M, Balk EM, Stel VS, Wright S, et al. GFR at Initiation of Dialysis and Mortality in CKD: A Meta-analysis. American journal of kidney diseases : the official journal of the National Kidney Foundation. 2012;59(6):829-40.
16. NHS Blood and Transplant. Kidney Activity 2015 [Available from: http://nhsbtmediaservices.blob.core.windows.net/organ-donation-assets/pdfs/kidney_activity.pdf.
17. Kerr M, Bray B, Medcalf J, O'Donoghue DJ, Matthews B. Estimating the financial cost of chronic kidney disease to the NHS in England. Nephrol Dial Transplant. 2012;27 Suppl 3:iii73-80.
18. Office for National Statistics. National Population Projections, 2010-Based Projections 2011 [Available from: http://www.ons.gov.uk/ons/rel/npp/national-population-projections/2010-based-projections/rep-2010-based-npp-results-summary.html.
19. Wang AY, Ninomiya T, Al-Kahwa A, Perkovic V, Gallagher MP, Hawley C, et al. Effect of hemodiafiltration or hemofiltration compared with hemodialysis on mortality and cardiovascular disease in chronic kidney failure: a systematic review and meta-analysis of randomized trials. Am J Kidney Dis. 2014;63(6):968-78.
20. Maduell F, Moreso F, Pons M, Ramos R, Mora-Macia J, Carreras J, et al. High-efficiency postdilution online hemodiafiltration reduces all-cause mortality in hemodialysis patients. J Am Soc Nephrol. 2013;24(3):487-97.

21. Ok E, Asci G, Toz H, Ok ES, Kircelli F, Yilmaz M, et al. Mortality and cardiovascular events in online haemodiafiltration (OL-HDF) compared with high-flux dialysis: results from the Turkish OL-HDF Study. Nephrol Dial Transplant. 2013;28(1):192-202.
22. Grooteman MP, van den Dorpel MA, Bots ML, Penne EL, van der Weerd NC, Mazairac AH, et al. Effect of online hemodiafiltration on all-cause mortality and cardiovascular outcomes. J Am Soc Nephrol. 2012;23(6):1087-96.
23. Penne EL, van der Weerd NC, Bots ML, van den Dorpel MA, Grooteman MP, Levesque R, et al. Patient- and treatment-related determinants of convective volume in post-dilution haemodiafiltration in clinical practice. Nephrol Dial Transplant. 2009;24(11):3493-9.
24. Mazairac AH, Blankestijn PJ, Grooteman MP, Penne EL, van der Weerd NC, den Hoedt CH, et al. The cost-utility of haemodiafiltration versus haemodialysis in the Convective Transport Study. Nephrol Dial Transplant. 2013;28(7):1865-73.
25. Levesque R, Marcelli D, Cardinal H, Caron ML, Grooteman MP, Bots ML, et al. Cost-Effectiveness Analysis of High-Efficiency Hemodiafiltration Versus Low-Flux Hemodialysis Based on the Canadian Arm of the CONTRAST Study. Applied health economics and health policy. 2015;13(6):647-59.
26. Vilar E, Wellsted D, Chandna SM, Greenwood RN, Farrington K. Residual renal function improves outcome in incremental haemodialysis despite reduced dialysis dose. Nephrol Dial Transplant. 2009;24(8):2502-10.
27. Fry AC, Singh DK, Chandna SM, Farrington K. Relative importance of residual renal function and convection in determining beta-2-microglobulin levels in high-flux haemodialysis and on-line haemodiafiltration. Blood purification. 2007;25(3):295-302.
28. Termorshuizen F, Dekker FW, van Manen JG, Korevaar JC, Boeschoten EW, Krediet RT, et al. Relative contribution of residual renal function and different measures of adequacy to survival in hemodialysis patients: an analysis of the Netherlands Cooperative Study on the Adequacy of Dialysis (NECOSAD)-2. J Am Soc Nephrol. 2004;15(4):1061-70.
29. Tattersall JE, Ward RA, group E. Online haemodiafiltration: definition, dose quantification and safety revisited. Nephrol Dial Transplant. 2013;28(3):542-50.
30. Colangelo PM, Welch DW, Rich DS, Jeffrey LP. Two methods for estimating body surface area in adult amputees. American journal of hospital pharmacy. 1984;41(12):2650-5.
31. Eknoyan G, Beck GJ, Cheung AK, Daugirdas JT, Greene T, Kusek JW, et al. Effect of dialysis dose and membrane flux in maintenance hemodialysis. The New England journal of medicine. 2002;347(25):2010-9.
32. Locatelli F, Martin-Malo A, Hannedouche T, Loureiro A, Papadimitriou M, Wizemann V, et al. Effect of membrane permeability on survival of hemodialysis patients. J Am Soc Nephrol. 2009;20(3):645-54.
33. Tattersall J, Canaud B, Heimburger O, Pedrini L, Schneditz D, Van Biesen W, et al. High-flux or low-flux dialysis: a position statement following publication of the Membrane Permeability Outcome study. Nephrol Dial Transplant. 2010;25(4):1230-2.
34. Donovan JL, Rooshenas L, Jepson M, Elliott D, Wade J, Avery K, et al. Optimising recruitment and informed consent in randomised controlled trials: the development and implementation of the Quintet Recruitment Intervention (QRI). Trials. 2016;17(1):283.
35. Donovan J, Mills N, Smith M, Brindle L, Jacoby A, Peters T, et al. Quality improvement report: Improving design and conduct of randomised trials by embedding them in qualitative research: ProtecT (prostate testing for cancer and treatment) study. Commentary: presenting unbiased information to patients can be difficult. BMJ (Clinical research ed. 2002;325(7367):766-70.
36. Rooshenas L, Elliott D, Wade J, Jepson M, Paramasivan S, Strong S, et al. Conveying Equipoise during Recruitment for Clinical Trials: Qualitative Synthesis of Clinicians' Practices across Six Randomised Controlled Trials. PLoS medicine. 2016;13(10):e1002147.
37. Donovan JL, de Salis I, Toerien M, Paramasivan S, Hamdy FC, Blazeby JM. The intellectual challenges and emotional consequences of equipoise contributed to the fragility of recruitment in six randomized controlled trials. Journal of clinical epidemiology. 2014;67(8):912-20.
38. Donovan JL, Paramasivan S, de Salis I, Toerien M. Clear obstacles and hidden challenges: understanding recruiter perspectives in six pragmatic randomised controlled trials. Trials. 2014;15:5.
39. Paramasivan S, Huddart R, Hall E, Lewis R, Birtle A, Donovan JL. Key issues in recruitment to randomised controlled trials with very different interventions: a qualitative investigation of recruitment to the SPARE trial (CRUK/07/011). Trials. 2011;12:78.

40. de Salis I, Tomlin Z, Toerien M, Donovan J. Using qualitative research methods to improve recruitment to randomized controlled trials: the Quartet study. Journal of health services research & policy. 2008;13 Suppl 3:92-6.
41. Strauss A, Corbin J. Grounded theory methodology. Handbook of qualitative research. . 171994. p. 273-85.
42. Wade J, Donovan JL, Lane JA, Neal DE, Hamdy FC. It's not just what you say, it's also how you say it: opening the 'black box' of informed consent appointments in randomised controlled trials. Social science & medicine (1982). 2009;68(11):2018-28.
43. Paramasivan S, Strong S, Wilson C, Campbell B, Blazeby JM, Donovan JL. A simple technique to identify key recruitment issues in randomised controlled trials: Q-QAT - Quanti-Qualitative Appointment Timing. Trials. 2015;16:88.
44. Gilg J, Caskey FJ, Fogarty D. UK Renal Registry 18th Annual Report: Chapter 1 UK Renal Replacement Therapy Incidence in 2014: National and Centre-specific Analyses. Nephron Clin Pract. 2016.
45. Rao A, Pitcher D, Fluck R, Kumwenda M. UK Renal Registry 17th Annual Report: Chapter 10 2013 Multisite Dialysis Access Audit in England, Northern Ireland and Wales and 2012 PD One Year Follow-up: National and Centre-specific Analyses. Nephron. 2015;129 Suppl 1:223-45.
46. Drummond MF, O'Brien B, Stoddart GL, Torrance GW. Methods for the economic evaluation of health care programmes. Oxford, UK: Oxford University Press; 1997.
47. Oates T, Cross J, Davenport A. Cost comparison of online haemodiafiltration with high-flux haemodialysis. Journal of nephrology. 2012;25(2):192-7.
48. Health Do. Reference Costs 2014-15. 2015.
49. Unit PSSR. Unit Costs of Health and Social Care 2015 2015 [Available from: http://www.pssru.ac.uk/project-pages/unit-costs/2015/index.php.
50. Hoch JS, Briggs AH, Willan AR. Something old, something new, something borrowed, something blue: a framework for the marriage of health econometrics and cost-effectiveness analysis. Health economics. 2002;11(5):415-30.
51. Lothgren M, Zethraeus N. Definition, interpretation and calculation of cost-effectiveness acceptability curves. Health economics. 2000;9(7):623-30.
52. Davies SJ, Phillips L, Naish PF, Russell GI. Quantifying comorbidity in peritoneal dialysis patients and its relationship to other predictors of survival. Nephrol Dial Transplant. 2002;17(6):1085-92.
53. Charlson ME, Pompei P, Ales KL, MacKenzie CR. A new method of classifying prognostic co-morbidity in longitudinal studies: development and validation. J Chron Dis. 1987;40(5):373-83.
54. Oppe M, Devlin NJ, van Hout B, Krabbe PF, de Charro F. A program of methodological research to arrive at the new international EQ-5D-5L valuation protocol. Value Health. 2014;17(4):445-53.
55. Hays RD, Kallich JD, Mapes DL, Coons SJ, Carter WB. Development of the kidney disease quality of life (KDQOL) instrument. Qual Life Res. 1994;3(5):329-38.
